# Three redescriptions in *Tintinnopsis* (Protista: Ciliophora: Tintinnina) from coastal waters of China, with cytology and phylogenetic analyses based on ribosomal RNA genes

**DOI:** 10.1186/s12866-020-02057-2

**Published:** 2020-12-14

**Authors:** Yang Bai, Rui Wang, Wen Song, Lifang Li, Luciana F. Santoferrara, Xiaozhong Hu

**Affiliations:** 1 College of Fisheries, & Key Laboratory of Mariculture, Ministry of Education, Ocean University of China, Qingdao, 266003 China; 2 Institute of Evolution and Marine Biodiversity, Ocean University of China, Qingdao, 266003 China; 3 Marine College, Shandong University, Weihai, 264209 China; 4 Department of Ecology and Evolutionary Biology and Department of Marine Sciences, University of Connecticut, One University Place, Stamford, CT 06901 USA

**Keywords:** Ciliary pattern, Ciliary tuft, Lorica, Non-monophyly

## Abstract

**Background:**

The taxonomy of tintinnine ciliates is vastly unresolved because it has traditionally been based on the lorica (a secreted shell) and it has only recently incorporated cytological and molecular information. *Tintinnopsis,* the most speciose tintinnine genus, is also the most problematic: it is known to be non-monophyletic, but it cannot be revised until more of its species are studied with modern methods.

**Results:**

Here, *T. hemispiralis* Yin, 1956, *T. kiaochowensis* Yin, 1956, and *T. uruguayensis* Balech, 1948, from coastal waters of China, were studied. Lorica and cell features were morphometrically investigated in living and protargol-stained specimens, and sequences of three ribosomal RNA (rRNA) loci were phylogenetically analyzed. The three species show a complex ciliary pattern (with ventral, dorsal, and posterior kineties and right, left, and lateral ciliary fields), but differ in lorica morphology, details of the somatic ciliature and rRNA gene sequences. *Tintinnopsis hemispiralis* is further distinguished by a ciliary tuft (a ribbon of very long cilia originated from the middle portion of the ventral kinety and extending out of the lorica) and multiple macronuclear nodules. Both *T. kiaochowensis* and *T. uruguayensis* have two macronuclear nodules, but differ in the number of somatic kineties and the position of the posterior kinety. Two neotypes are fixed for *T. hemispiralis* and *T. kiaochowensis* to stabilize the species names objectively, mainly because of the previous unavailability of type materials. By phylogenetic analysis and comparison with closely-related species, we infer that the ciliary tuft and details such as the commencement of the rightmost kinety in the lateral ciliary field are synapomorphies that may help clarify the systematics of *Tintinnopsis*-like taxa.

**Conclusion:**

The redescriptions of three poorly known *Tintinnopsis* species, namely *T. hemispiralis*, *T. kiaochowensis*, and *T. uruguayensis* firstly revealed their ciliary patterns and rRNA sequences. This study expands knowledge and database of tintinnines and helps in identifying potential synapomorphies for future taxonomic rearrangements.

## Background

Ciliated protists are among the most diverse and numerically important members of microzooplankton, and act as a trophic link in the microbial food web of aquatic ecosystems [1–5]. In particular, tintinnine ciliates are conspicuous due to the diversity of loricae produced by their cell propers. Tintinnines have been of great interest in the field of protistology because they (i) display distinct patterns of diversity and biogeography [6, 7]; (ii) serve as bioindicators of water quality and hydrological circulation [8–11]; (iii) are prey for fish larvae and other small metazoans [12, 13]; and (iv) can leave fossilized loricae that are useful in evolutionary studies [14, 15].

There are approximately 1000 extant tintinnine species classified almost entirely based on the shape and size of their loricae [16–21]. However, it is widely recognized that lorica features alone have shortcomings for determining taxonomic affiliations in this group of ciliates [22, 23]. In some species, laboratory cultures have provided clear evidences that the lorica is polymorphic in response to environmental factors or in different stages of the life cycle [24]. More recently, DNA sequencing of several closely-related species has revealed examples of polymorphic and cryptic species [25, 26]. Thus, the current lorica-based taxonomy does not allow estimating tintinnine diversity accurately, and it does not provide a natural classification. Accordingly, several studies have incorporated more informative characters, namely, cytological and/ or molecular data, in tintinnine systematics (e.g., [27–36]). Still, cell characters and DNA sequences are only known for about 3 and 10% of the described tintinnine morphospecies, respectively (e.g., [22, 37]), and considerable efforts are needed to increase the availability of these types of information.

Arguably the most problematic taxon in tintinnine taxonomy is the genus *Tintinnopsis* Stein, 1867. This genus is known as artificial, given that it includes at least five distinct ciliary patterns [22, 33, 38] and more than ten clades that are non-monophyletic in rDNA sequence analyses [39]. Out of the about 140 *Tintinnopsis*-like morphospecies [19–21], about 60 have been recorded in China seas (e.g. [21, 40–44]), but only a few count with ciliature and/or sequence data [34, 36, 45–48]. Overall, *Tintinnopsis* will need subdivision once its type, *T. beroidea*, and other species are studied with modern methods [22, 38, 39].

The present study investigates the morphology and molecular phylogeny of three *Tintinnopsis* species, namely, *T. hemispiralis* Yin, 1956, *T. kiaochowensis* Yin, 1956, and *T. uruguayensis* Balech, 1948, which were collected from coastal waters of China. This work includes observations of specimens in vivo and after protargol staining as well as phylogenetic analyses of ribosomal RNA gene markers based on recommendations for tintinnine taxonomy [23] and common practices for other ciliates [49]. The aims of the present study are to combine lorica, cell proper, and molecular data in three *Tintinnopsis* species and to compare them with related taxa in order to find potential diagnostic features relevant in this problematic tintinnine taxon.

## Zoobank registration

The ZooBank registration number of the present work is: urn:lsid:zoobank.org:pub:38490F0B-183F-45AE-A053-80FCA6799716.

## Results

**Order Choreotrichida Small and Lynn, 1985**

**Suborder Tintinnina Kofoid and Campbell, 1929**

**Genus** ***Tintinnopsis*** **Stein, 1867**

**Fig. 1:**
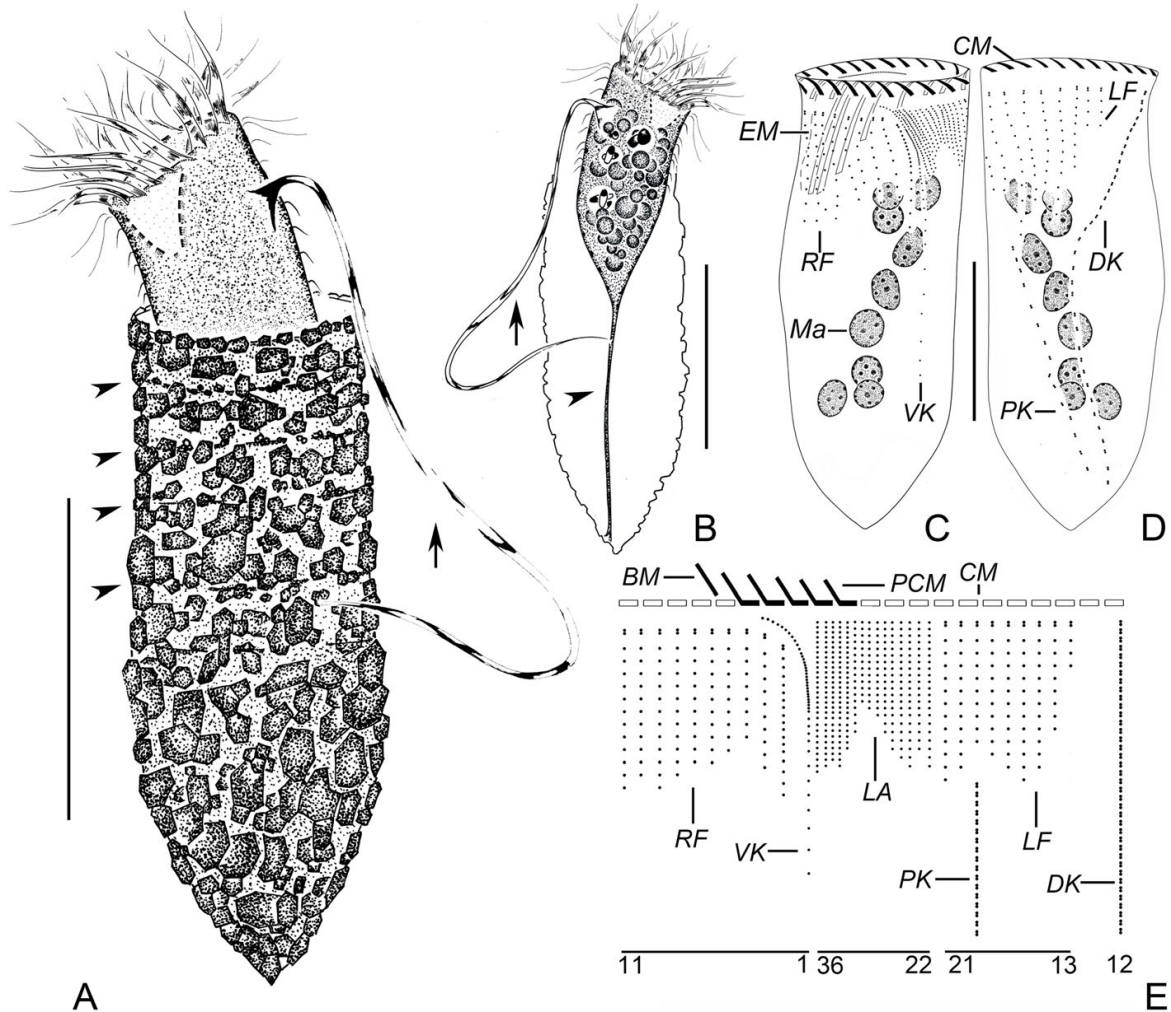
Line drawings of *Tintinnopsis hemispiralis* in vivo (**a**, **b**) and after protargol staining (**c**–**e**) (from authors’ own work). **a** Lateral view of a representative individual; arrow denotes the ciliary tuft; arrowheads mark the spiral striations on the collar portion of lorica. **b** Cell characters; arrow denotes the ciliary tuft; arrowhead shows peduncle. **c**, **d** Ventral (**c**) and dorsal (**d**) views of the same specimen, showing ciliary pattern and macronuclear nodules. **e** Kinetal map of a morphostatic specimen. BM, buccal membranelle; CM, collar membranelle; DK, dorsal kinety; EM, endoral membrane; LA, lateral ciliary field; LF, left ciliary field; Ma, macronuclear nodule; PCM, prolonged collar membranelle; PK, posterior kinety; RF, right ciliary field; VK, ventral kinety. Scale bars = 75 μm (**a**, **b**), 30 μm (**c**, **d**)

**Fig. 2:**
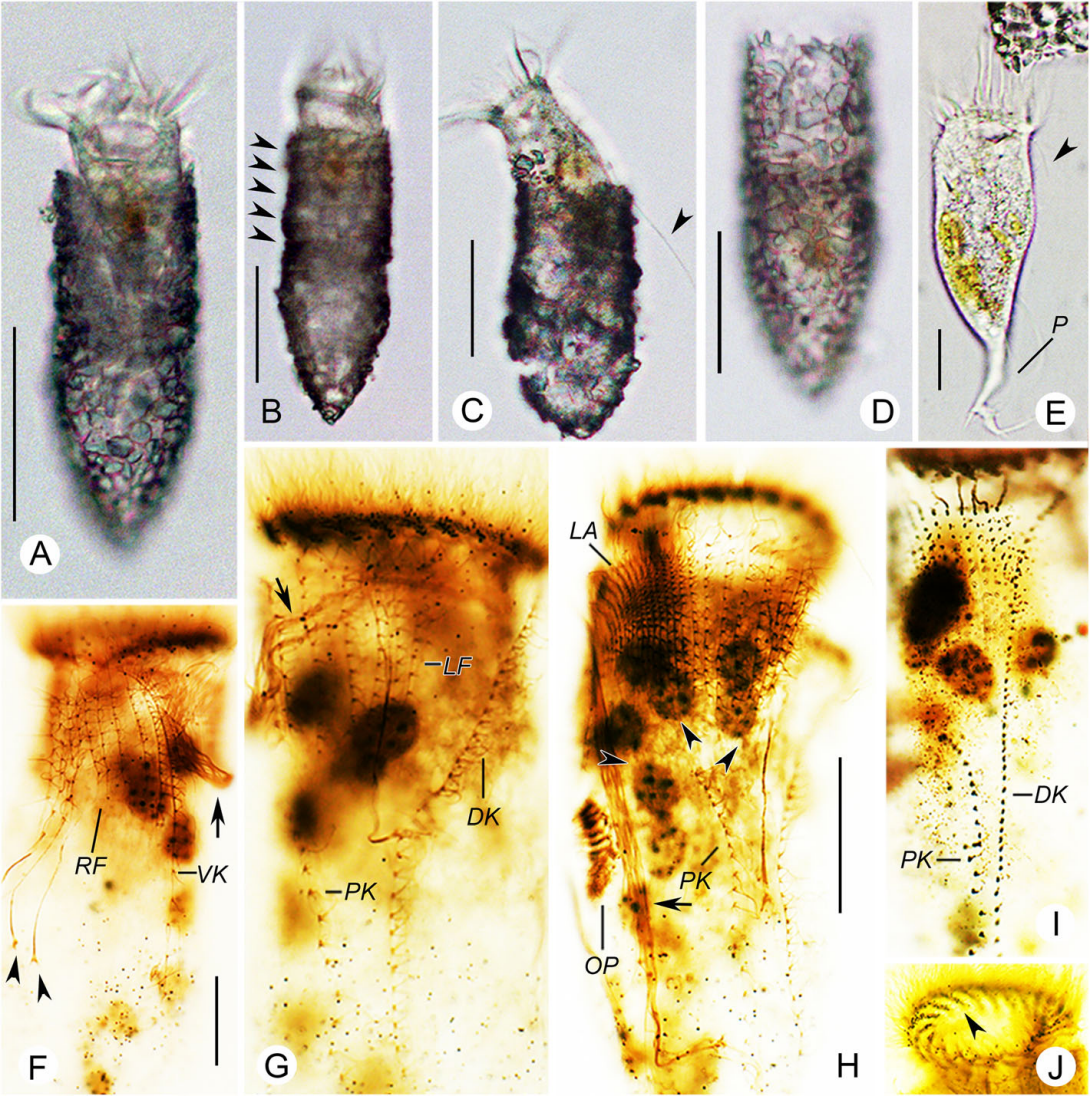
Photomicrographs of *Tintinnopsis hemispiralis* in vivo (**a**–**e**) and after protargol staining (**f**–**j**). **a** Lateral view of a representative individual. **b** Arrowheads show the spiral striations on lorica. **c** Fully extended individual with broken lorica; arrowhead shows the ciliary tuft. **d** Lorica of another individual. **e** Cell proper that abandoned the lorica; arrowhead shows elongated anterior cilia. **f** Ventral kinety and right ciliary fields; arrow shows the ciliary tuft; arrowheads indicate the nodules of the thick argyrophilic fibers. **g** Dorsal side, showing the left ciliary field, dorsal kinety, and posterior kinety; arrow marks the ciliary tuft. **h** Lateral side of an early divider; arrowheads mark macronuclear nodules; arrow shows the ciliary tuft. **i** Posterior kinety and dorsal kinety. **j** Arrowhead marks collar membranelles. DK, dorsal kinety; LA, lateral ciliary field; LF, left ciliary field; OP, oral primordium; PK, posterior kinety; RF, right ciliary field; P, peduncle; VK, ventral kinety. Scale bars = 75 μm (**a**, **c**), 60 μm (**b**, **d**), 25 μm (**e**), 15 μm (**f**), 20 μm (**h**)

***Tintinnopsis hemispiralis*** **Yin, 1956** (Figs. 1a–e, 2a–j; Table 1).

**Table 1:** Morphometric data of *Tintinnopsis hemispiralis*, *T. kiaochowensis*, and *T. uruguayensis* (measurements in μm). Lorica data are based on live specimens, and other data are based on protargol-stained specimens

Characters	Species name	Min	Max	Mean	M	SD	CV	N
Lorica, total length	*T. hemispiralis*	143	182	161.1	161	12.9	8.0	15
	*T. kiaochowensis*	79	112	89.9	90	8.2	9.1	12
	*T. uruguayensis*	50	73	62.3	62	7.7	12.4	15
Lorica, bowl width	*T. hemispiralis*	49	66	55.9	57	5.1	9.2	15
	*T. kiaochowensis*	57	81	66.7	65	7.4	11.0	12
	*T. uruguayensis*	25	41	32.6	32	4.5	13.7	15
Lorica, bowl length	*T. hemispiralis*	68	88	77.8	76	6.6	8.5	15
	*T. kiaochowensis*	43	70	51.3	49	7.2	14.1	12
	*T. uruguayensis*	32	52	39.0	39	5.7	14.6	13
Lorica, collar length	*T. hemispiralis*	64	97	83.3	86	8.4	10.1	15
	*T. kiaochowensis*	32	46	38.7	38	4.6	12.0	12
	*T. uruguayensis*	11	22	16.6	16	3.2	19.4	15
Lorica, opening diameter	*T. hemispiralis*	45	59	52.6	54	4.5	8.6	15
	*T. kiaochowensis*	44	71	55.1	53	7.5	13.6	12
	*T. uruguayensis*	24	42	33.1	33	5.4	16.4	15
Lorica, length: opening diameter, ratio	*T. hemispiralis*	2.9	3.2	3.1	3.1	0.1	2.4	15
	*T. kiaochowensis*	1.4	2.1	1.6	1.6	0.2	10.5	12
	*T. uruguayensis*	1.6	2.3	1.9	1.9	0.2	8.2	15
Lorica, narrowed portion diameter	*T. kiaochowensis*	38	63	48.8	47	7.7	15.9	12
	*T. uruguayensis*	17	29	24.2	25	3.7	15.5	15
Lorica, total length: narrowed portion diameter	*T. kiaochowensis*	1.4	2.2	1.9	2.0	0.3	16.2	12
	*T. uruguayensis*	1.8	3.2	2.6	2.6	0.4	16.0	15
Cell proper, length	*T. hemispiralis*	65	119	95.1	95	15.0	15.8	15
	*T. kiaochowensis*	46	65	55.9	58	5.8	10.4	12
	*T. uruguayensis*	25	56	31.5	30	7.4	23.5	15
Cell proper, width	*T. hemispiralis*	31	58	44.5	47	8.3	18.6	15
	*T. kiaochowensis*	38	64	45.5	42	6.5	14.4	12
	*T. uruguayensis*	18	28	22.5	22	3.0	13.3	15
Macronuclear nodules, number	*T. hemispiralis*	7	11	9.2	9	1.0	11.0	15
	*T. kiaochowensis*	2	2	2.0	2	0.0	0.0	12
	*T. uruguayensis*	2	2	2.0	2	0.0	0.0	15
Macronuclear nodules, length	*T. hemispiralis*	5	10	8.7	9	1.3	14.9	15
	*T. kiaochowensis*	15	22	18.4	18	2.4	12.8	12
	*T. uruguayensis*	6	14	8.3	7	2.3	27.2	15
Macronuclear nodules, width	*T. hemispiralis*	5	9	7.4	7	0.9	12.3	15
	*T. kiaochowensis*	12	17	14.3	15	1.6	11.3	12
	*T. uruguayensis*	4	10	5.8	5	1.8	31.4	15
Anterior cell end to anterior macronucleus nodule, distance	*T. hemispiralis*	17	21	19.1	19	1.8	9.4	15
	*T. kiaochowensis*	11	24	19.2	18	4.1	21.6	12
	*T. uruguayensis*	4	9	6.3	6	1.4	22.1	15
Ventral kinety, length	*T. hemispiralis*	39	66	56.6	55	5.2	9.3	15
	*T. kiaochowensis*	22	37	32.3	34	4.7	14.5	12
	*T. uruguayensis*	14	35	19.1	17	5.9	31.0	15
Ventral kinety, number of kinetids	*T. hemispiralis*	41	61	52.5	52	5.1	9.7	15
	*T. kiaochowensis*	43	56	48.6	48	4.0	8.2	12
	*T. uruguayensis*	17	28	20.3	21	2.3	11.3	15
Ventral kinety, distance to anterior end of cell	*T. hemispiralis*	4	8	5.5	5	0.8	15.3	15
	*T. kiaochowensis*	3	5	4.1	4	0.5	12.6	12
	*T. uruguayensis*	2	3	2.3	2	0.5	20.9	15
Dorsal kinety, length	*T. hemispiralis*	66	97	88.8	90	6.1	6.9	15
	*T. kiaochowensis*	29	53	43.8	43	6.5	14.8	12
	*T. uruguayensis*	21	41	26.5	24	5.5	20.8	15
Dorsal kinety, number of kinetids	*T. hemispiralis*	35	56	46.5	43	4.7	10.2	15
	*T. kiaochowensis*	25	37	31.2	31	4.4	14.3	12
	*T. uruguayensis*	17	29	21.1	20	3.5	16.7	15
Dorsal kinety, distance to right ciliary field	*T. hemispiralis*	4	6	4.5	4	0.6	14.1	15
	*T. kiaochowensis*	4	6	4.7	5	0.7	14.0	12
	*T. uruguayensis*	2	3	2.2	2	0.4	18.2	15
Dorsal kinety, distance to left ciliary field	*T. hemispiralis*	8	14	10.4	11	1.1	10.8	15
	*T. kiaochowensis*	11	21	13.2	13	2.6	19.7	12
	*T. uruguayensis*	2	5	3.2	3	0.7	21.8	15
Dorsal kinety, distance to anterior end of cell	*T. hemispiralis*	4	7	4.9	5	1.0	20.9	15
	*T. kiaochowensis*	3	6	4.4	5	0.9	20.4	12
	*T. uruguayensis*	2	3	2.3	2	0.5	20.9	15
Posterior kinety, length	*T. hemispiralis*	37	58	45.9	45	5.3	11.5	15
	*T. kiaochowensis*	21	29	25.5	27	3.1	12.2	11
	*T. uruguayensis*	11	22	13.7	12	2.9	21.0	15
Posterior kinety, number of kinetids	*T. hemispiralis*	11	22	17.0	17	2.7	15.7	15
	*T. kiaochowensis*	11	18	15.4	15	2.0	12.8	11
	*T. uruguayensis*	7	9	8.0	8	0.5	6.7	15
Posterior kinety, distance to anterior end of cell	*T. hemispiralis*	29	41	34.9	34	3.4	9.7	15
	*T. kiaochowensis*	28	49	36.0	35	5.3	14.6	12
	*T. uruguayensis*	12	21	15.5	15	2.5	16.1	15
Right ciliary field, number of kineties	*T. hemispiralis*	9	11	9.7	9	0.8	8.4	15
	*T. kiaochowensis*	10	13	11.4	12	0.9	7.9	12
	*T. uruguayensis*	7	8	7.3	7	0.5	6.3	15
Longest kinety in right field, length	*T. hemispiralis*	16	26	19.7	19	2.5	12.7	15
	*T. kiaochowensis*	19	28	24.3	25	2.5	10.4	12
	*T. uruguayensis*	7	14	10.3	10	2.2	20.9	15
Longest kinety in right field, number of kinetids	*T. hemispiralis*	15	19	16.6	16	1.0	5.9	15
	*T. kiaochowensis*	11	15	12.4	12	1.1	8.7	12
	*T. uruguayensis*	7	9	7.4	7	0.6	8.5	15
Shortest kinety in right field, length	*T. hemispiralis*	6	16	10.7	11	2.0	18.5	15
	*T. kiaochowensis*	9	17	13.5	14	1.8	13.6	12
	*T. uruguayensis*	3	6	4.5	4	0.9	20.5	15
Shortest in right field, number of kinetids	*T. hemispiralis*	6	11	8.6	8	2.2	25.2	15
	*T. kiaochowensis*	6	7	6.5	7	0.5	8.0	12
	*T. uruguayensis*	2	3	2.8	3	0.4	14.8	15
Left ciliary field, number of kineties	*T. hemispiralis*	9	12	9.8	9	1.0	10.3	15
	*T. kiaochowensis*	9	11	9.9	10	0.7	6.7	12
	*T. uruguayensis*	6	8	6.6	6	0.8	12.5	15
Longest kinety in left field, length	*T. hemispiralis*	17	28	23.3	24	3.3	14.0	15
	*T. kiaochowensis*	11	16	12.5	12	1.4	11.6	12
	*T. uruguayensis*	7	11	9.1	9	1.1	12.3	15
Longest kinety in left field, number of kinetids	*T. hemispiralis*	11	14	12.7	13	0.8	6.3	15
	*T. kiaochowensis*	8	9	8.8	9	0.5	5.2	12
	*T. uruguayensis*	6	8	7.3	7	0.6	8.2	15
Shortest kinety in left field, length	*T. hemispiralis*	4	8	5.8	6	1.3	22.8	15
	*T. kiaochowensis*	4	7	5.8	6	1.0	17.7	12
	*T. uruguayensis*	3	6	4.6	4	0.9	19.8	15
Shortest kinety in left field, number of kinetids	*T. hemispiralis*	3	5	3.7	4	0.6	15.9	15
	*T. kiaochowensis*	3	4	3.6	4	0.5	14.4	12
	*T. uruguayensis*	2	2	2.0	2	0.0	0.0	15
Lateral ciliary field, number of kineties	*T. hemispiralis*	11	20	15.4	15	2.3	7.7	15
	*T. kiaochowensis*	13	19	15.9	15	1.8	11.5	12
	*T. uruguayensis*	9	16	11.9	11	2.3	19.1	15
Lateral ciliary field, length of the longest kinety	*T. hemispiralis*	9	17	12.4	12	1.5	12.5	15
	*T. kiaochowensis*	22	33	28.9	31	4.0	13.7	12
	*T. uruguayensis*	9	14	11.8	12	1.6	13.7	15
Lateral ciliary field, length of the shortest kinety	*T. hemispiralis*	4	8	6.3	6	1.0	16.5	15
	*T. kiaochowensis*	9	19	13.4	14	2.7	20.5	12
	*T. uruguayensis*	7	11	9.5	10	1.4	14.9	15
Kineties in ciliary field, distance to anterior end of cell	*T. hemispiralis*	7	12	9.3	9	1.5	16.0	15
	*T. kiaochowensis*	6	13	9.4	9	1.9	20.0	12
	*T. uruguayensis*	2	3	2.3	2	0.5	20.9	15
Adoral zone of membranelles, diameter	*T. hemispiralis*	27	57	40.7	40	8.5	20.8	15
	*T. kiaochowensis*	33	52	42.9	42	5.2	12.0	12
	*T. uruguayensis*	12	21	17.0	17	1.9	11.1	15
Collar membranelles, number	*T. hemispiralis*	20	22	21.3	21	0.7	3.4	15
	*T. kiaochowensis*	16	18	16.3	16	0.5	2.8	12
	*T. uruguayensis*	18	19	18.2	18	0.4	2.3	15
Buccal membranelle, number	*T. hemispiralis*	1	1	1.0	1	0.0	0.0	15
	*T. kiaochowensis*	1	1	1.0	1	0.0	0.0	12
	*T. uruguayensis*	1	1	1.0	1	0.0	0.0	15
Prolonged membranelles, number	*T. hemispiralis*	4	5	4.7	5	0.5	10.5	15
	*T. kiaochowensis*	3	3	3.0	3	0.0	0.0	12
	*T. uruguayensis*	3	4	3.3	3	0.5	14.0	15

*Abbreviations: CV* Coefficient of variation in %, *M* Median, *Max* Maximum, *Mean* Arithmetic mean, *Min* Minimum, *N* Number of specimens examined, *SD* Standard deviation

### Terminology

*Tintinnopsis hemispiralis* possesses a cluster of extremely long cilia that has only been reported for *Tintinnopsis subacuta* [50]. This character is here defined as follows.

*Ciliary tuft.* An extraordinary long tuft of cilia originated from densely arranged kinetids in the middle portion of the ventral kinety.

### Improved diagnosis (based on the type and neotype populations)

Lorica 88–182 μm long, comprising a cylindrical, spiraled collar and an obconical bowl. Opening 34–59 μm in diameter. Cell proper elongate, obconical when fully extended, size in vivo 80–125 × 30–55 μm. Seven to 11 moniliform macronuclear nodules. On average 21 collar membranelles, of which four or five elongate into buccal cavity; one buccal membranelle. Ventral kinety composed of about 53 monokinetids, commences anteriorly to the second kinety of right ciliary field. Ciliary tuft about 150–250 μm long. Right and left ciliary fields consist of about ten kineties each. Lateral ciliary field comprises on average 15 kineties. Dorsal kinety composed of about 47 dikinetids. Posterior kinety with about 17 dikinetids, positioned below left ciliary field.

### Deposition of neotype and other voucher materials

A protargol slide including the neotype (Fig. 2f, g) was deposited in the Laboratory of Protozoology, Institute of Evolution and Marine Biodiversity, Ocean University of China (registration number: BY201805280101). One additional protargol slide was deposited in the same collection (registration number: BY201805280102).

### Redescription based on the Ningde population

Lorica 143–182 μm long, comprises a cylindrical, truncated collar and an obconical bowl (Figs. 1a, 2a–d). Opening 45–59 μm across. Ratio of lorica length to opening diameter 2.9–3.2:1. Collar 64–97 μm long, with three to five inconspicuous spiraled striations (Figs. 1a, 2b). Bowl often slightly wider than opening (49–66 μm in diameter), about 68–88 μm long, with a posterior angle of 45° (Figs. 1a, 2b, c, d). Wall of lorica heterogeneously agglutinated with mineral particles: collar slightly less agglutinated than bowl because adhered particles sparser and thinner (Figs. 1a, 2b, c, d).

Cell proper 80–125 μm long and 30–55 μm wide in vivo in fully extended, 65–119 μm long and 31–58 μm wide protargol preparations (Figs. 1b, 2e). Posterior portion of cell proper narrows gradually forming a peduncle with a branched posterior end, which is about 60–110 μm long and attaches to bottom of lorica (Figs. 1b, 2d, e). Seven to 11 moniliform macronuclear nodules, each about 5–10 long and 5–9 μm wide; anterior nodule 17–21 μm posterior to the anterior cell end in protargol-stained specimens (Figs. 1c, d, 2f, i, j). Micronuclei, striae, tentaculoids, accessory combs, a contractile vacuole, a cytopyge, and capsules not observed. Movement by irregular swimming with rotation about main cell axis.

Somatic ciliary pattern complex, that is, ventral kinety, dorsal kinety, posterior kinety, right ciliary field, left ciliary field, and lateral ciliary field present (Figs. 1c–e, 2f–i). Ventral kinety begins anteriorly to the second kinety of right ciliary field, about 4–8 μm below the anterior end of cell, goes around right ciliary field from left side before parallel to kineties of ciliary field posteriorly; 39–66 μm long, with 41–61 monokinetids, composed of three portions: (1) anterior portion comprised of eight to 14 kinetids about 0.5–1 μm apart; (2) middle portion consisting of 16–24 more densely arranged kinetids (with no measurable gap) with long cilia and forming the ciliary tuft, about 150–250 μm long in vivo; (3) posterior portion containing sparsely arranged monokinetids (more than 1 μm apart), extending posteriorly and terminating at about two thirds to three fourths of cell (Figs. 1a–c, e, 2c, f, g). Right ciliary field consists of 9–11 kineties, kineties about 2–5 μm away from their neighbors; each kinety has 5 to 18 monokinetids and one anterior dikinetid; kinetids of first kinety more densely arranged than those in the remaining kineties; all kineties commence at the same level (about 9 μm below the anterior end of cell), except for the first kinety that starts about 2 μm posteriorly to other kineties (Figs. 1c, e, 2f). Left ciliary field with 9–12 kineties, begins about 9 μm below the anterior end of cell, kineties about 2–5 μm away from their neighbors, composed of one anterior dikinetid and 2–13 monokinetids each; the leftmost two or three kineties always shorter, each only including three to five kinetids (Figs. 1c–e, 2g–i). The anterior basal bodies of dikinetids in left and right ciliary fields bear elongated cilia, about 20 μm long from life and 10 μm long after protargol staining while the cilia on posterior basal bodies are similar to ones on monokinetid in length, about 3 μm long after protargol staining (Figs. 1a, b, 2e, i). Lateral ciliary field commences about 9 μm posteriorly to the anterior end of cell, comprises 11–20, relatively densely arranged monokinetidal kineties; kineties in middle region always shorter than those at both ends of field (i.e., including only half the number of kineties); cilia about 3 μm long after silver staining (Figs. 1d, e, 2g, h). Dorsal kinety about 66–97 μm in length and consisting of 35–56 dikinetids, commences about 5 μm posteriorly to anterior cell end, about 5 and 10 μm away from right and left ciliary field, respectively; only the posterior basal body bearing a cilium about 8–10 μm long after protargol staining (Figs. 1d, e, 2g, i). Posterior kinety 37–58 μm long and consisting of 11–22 dikinetids, commences posteriorly to right portion of left ciliary field, with 29–41 μm away from the anterior cell end and curves rightwards; only the posterior basal body bearing a cilium about 8–10 μm long after protargol staining (Figs. 1d, e, 2g–i).

Adoral zone of membranelles composed of 20–22 collar membranelles with, four or five of which extend into buccal cavity with longest bases about 30 μm; cilia in collar membranelles about 25–35 μm in length; polykinetid structures could not be recognized (Figs. 1a–e, 2a–e, j). Single buccal membranelle within buccal cavity, with polykinetid about 40 μm long (Fig. 1c, e). Argyrophilic fibers originate in the proximal portions of the elongated collar membranelles and the buccal membranelle, and extend posteriorly; three thick fibers commencing from the middle of cell below right ciliary field and extending towards anterior part of cell; ends not observed due to insufficient staining (Fig. 2f). Endoral membrane consisting of a single row of basal bodies, extends in a semicircle across the peristomial field and right wall of buccal cavity (Fig. 1c). An early divider was observed with the oral primordium posterior to the ventral kinety and lateral ciliary field (Fig. 2h).

**Fig. 3:**
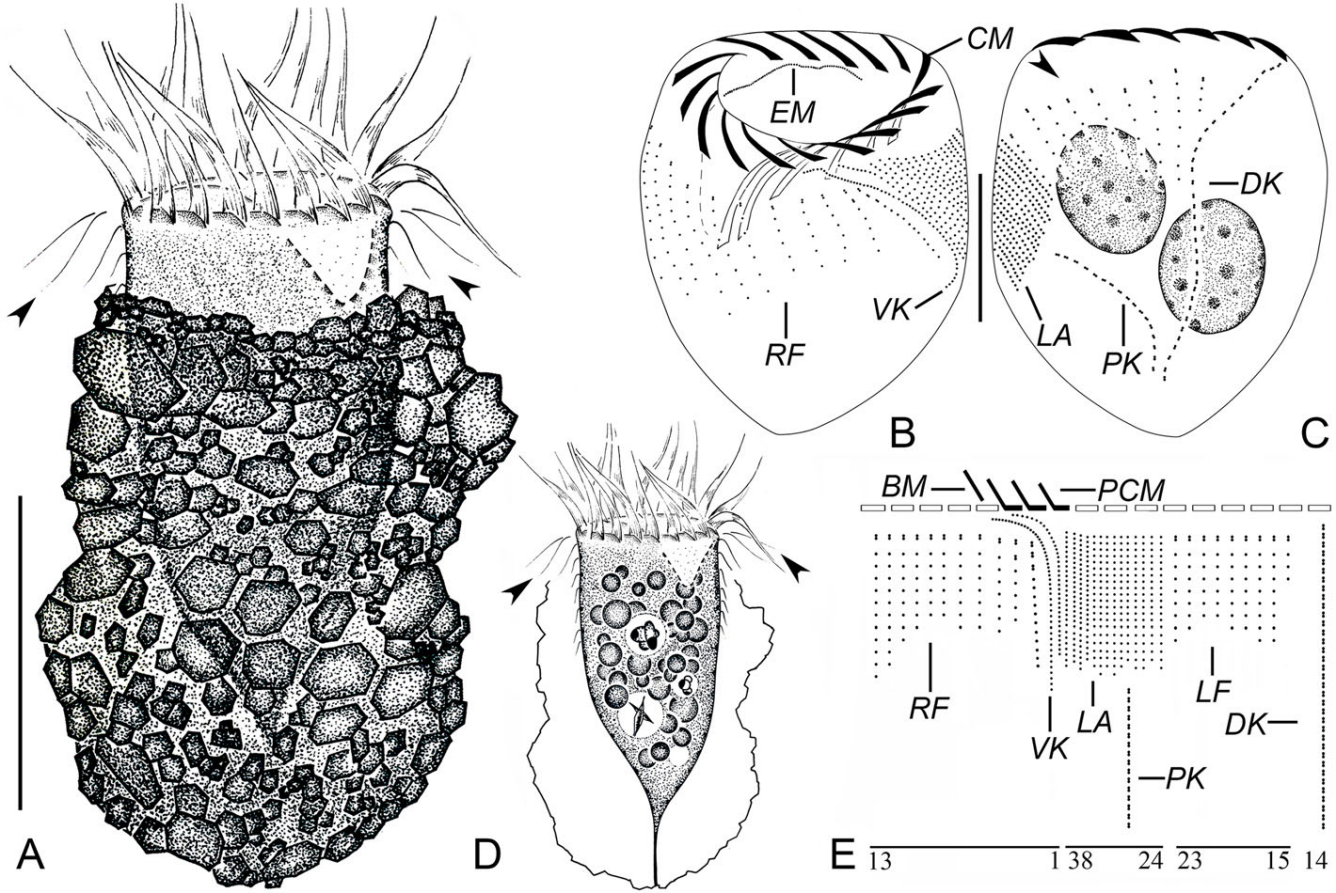
Line drawings of *Tintinnopsis kiaochowensis* in vivo (**a**, **d**) and after protargol staining (**b**, **c**, **e**) (from authors’ own work). **a** Lateral view of a representative individual; arrowheads mark elongated anterior cilia of right and left ciliary field. **b**, **c**, Ventral (**b**) and dorsal (**c**) views of the same specimen, showing ciliary pattern and macronuclear nodules; arrowhead denotes the left ciliary field. **d** Cell features; arrowheads mark elongated anterior cilium of the right and left ciliary fields; arrow shows peduncle. **e** Kinetal map of a morphostatic specimen. BM, buccal membranelle; CM, collar membranelle; DK, dorsal kinety; LA, lateral ciliary field; LF, left ciliary field; PCM, prolonged collar membranelle; PK, posterior kinety; RF, right ciliary field; VK, ventral kinety. Scale bars = 50 μm (**a**), 20 μm (**b**, **c**), 40 μm (**d**)

**Fig. 4:**
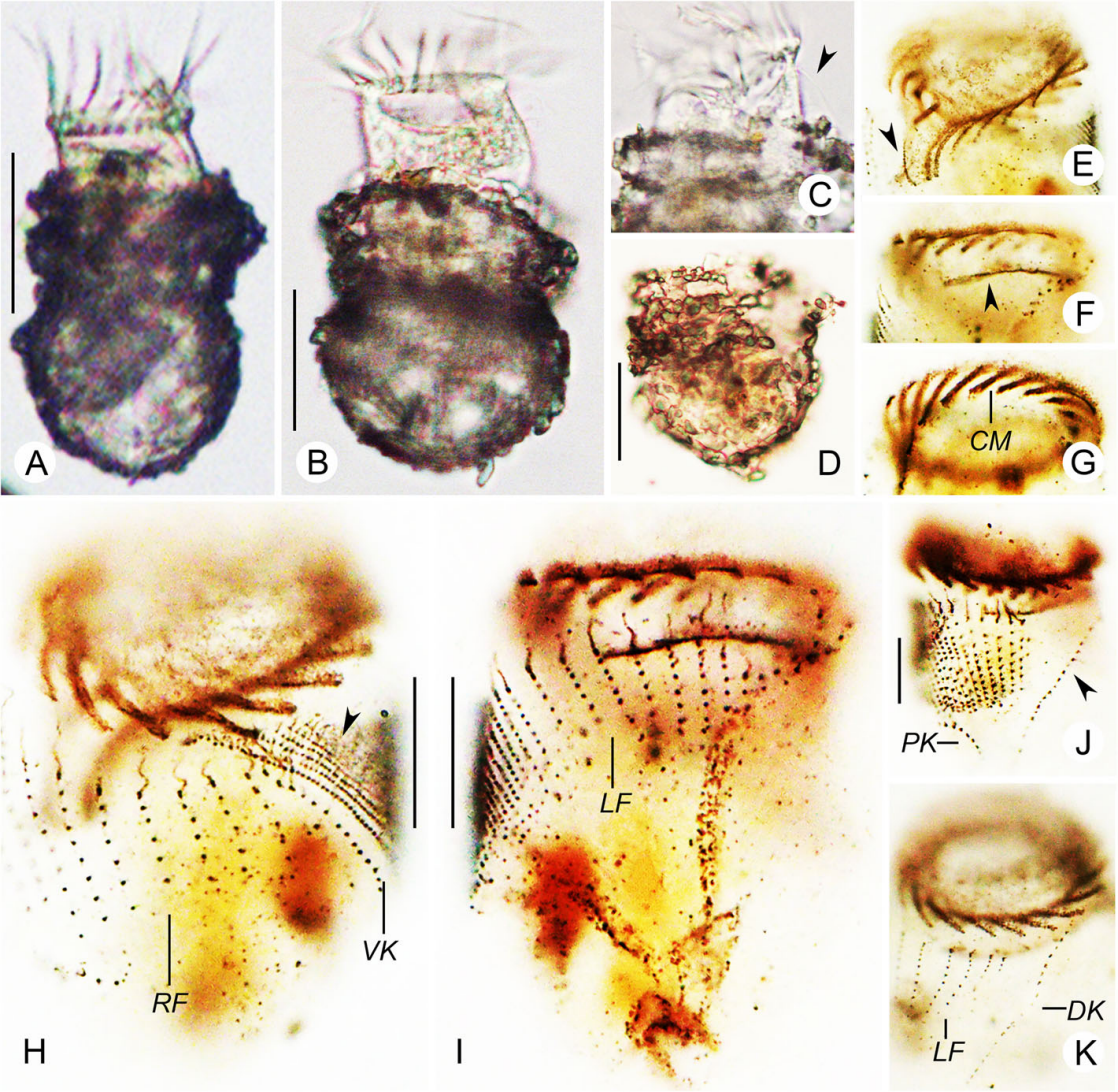
Photomicrographs of *Tintinnopsis kiaochowensis* in vivo (**a**–**d**) and after protargol staining (**e**–**k**). **a** Lateral view of a representative individual. **b** Different individual showing lorica variation. **c** Elongated anterior cilia of the right and left ciliary fields (arrowhead). **d** Pressed lorica showing aligned particles. **e**, **f** Arrowheads mark endoral membrane. **g** Collar membranelles. **h** Ventral side; arrowhead shows the lateral ciliary field. **i** Dorsal side of the same specimen as in (**h**). **j** Dorsal (arrowhead) and posterior kinety. **k** Subapical view, showing the left ciliary field and dorsal kinety. CM, collar membranelle; DK, dorsal kinety; LF, left ciliary field; PK, posterior kinety; RF, right ciliary field; VK, ventral kinety. Scale bars = 45 μm (**a**, **b**, **d**), 15 μm (**h**, **i**), 20 μm (**j**)

***Tintinnopsis kiaochowensis*** **Yin, 1956** (Figs. 3a–e, 4a–k; Table 1).

### Improved diagnosis (based on the type and neotype populations)

Lorica 79–112 μm in length, composed of an irregular collar and an ellipsoidal bowl with a rounded posterior end, both separated by a constriction. Opening 30–71 μm in diameter. Cell proper obconical when fully extended, size in vivo about 60–95 × 35–50 μm. Two ellipsoidal macronuclear nodules. On average 16 collar membranelles, three of which extend into buccal cavity; one buccal membranelle. Ventral kinety with an average of 49 densely arranged monokinetids. Right, left, and lateral ciliary fields include, on average, 11, 10, and 16 kineties, respectively. Dorsal kinety composed of about 31 dikinetids. Posterior kinety composed of about 15 dikinetids, positioned below lateral ciliary field.

### Deposition of neotype and other voucher materials

A protargol slide including the neotype (Fig. 4h, i) was deposited in the Laboratory of Protozoology, Institute of Evolution and Marine Biodiversity, Ocean University of China (registration number: BY201805280201). One additional protargol slide was deposited in the same collection (registration number: BY201805280202).

### Redescription based on the Ningde population

Lorica 79–112 μm in length, composed of an irregular collar and an ellipsoidal bowl (Figs. 3a, 4a–c). Opening 44–71 μm in diameter; rim irregular. Ratio of lorica length to opening diameter 1.4–2:1. Collar 32–46 μm high, not flaring at the opening margin, occasionally slightly layered because of agglutinated particles arranged in horizontal rows (Figs. 3a, 4a–c). Region between collar and bowl constricted, about 38–63 μm in diameter (Figs. 3a, 4a, b). Bowl about 43–70 μm long and 57–83 μm across. Posterior end usually rounded to bluntly tapered (Figs. 3a, 4a, b).

Cell proper about 60–95 μm long and 35–50 μm wide from life when it is fully extended, 46–65 μm long and 38–64 μm wide in protargol preparation. Posterior cell portion narrows successively forming a peduncle about 25 μm long and attached to the bottom of lorica (Figs. 3a, d, 4b). Two ellipsoidal macronuclear nodules, each about 15–22 × 12–17 μm in protargol-stained specimens; anterior nodule 11–24 μm from the anterior cell end (Fig. 3c). Micronuclei, striae, tentaculoids, accessory combs, contractile vacuole, cytopyge, and capsules not observed. Locomotion by rotation about main cell axis.

Somatic ciliary pattern complex, that is, ventral kinety, dorsal kinety, posterior kinety, right ciliary field, left ciliary field, and lateral ciliary field present (Figs. 3b, c, e, 4h–k). Ventral kinety 22–37 μm long, commences anteriorly to third or fourth kinety of right ciliary field and about 4 μm below the anterior cell end, anterior third curves leftwards before extending parallel to kineties of lateral ciliary field posteriorly; 43–56 densely arranged monokinetids (Figs. 3b, e, 4h). Right ciliary field consists of 10–13 kineties about 2–5 μm away from each other, the space between the leftmost five to six kineties wider than others; all kineties commence 6–13 μm below the anterior end of cell; composed of 5–14 widely spaced monokinetids and one anterior dikinetid, except for: (i) the first kinety almost parallel to ventral kinety, with two or three anterior dikinetids and eight to 12 monokinetids, more densely arranged than other kineties in right ciliary field; and (ii) the second kinety parallel to rest of kineties, with an angle of about 20° with the first kinety, including four or five monokinetids and two anterior dikinetids (Figs. 3b, e, 4h). Left ciliary field consists of 9–11 kineties 2–5 μm away from each other, each kinety commences 6–13 μm posteriorly to the anterior cell end and comprises of two to eight monokinetids and one anterior dikinetid; the number of kinetids of leftmost kinety always minimum (i.e., three or four). The anterior basal bodies of dikinetids in left and right ciliary field bear elongated cilia, about 15 μm long from life and 5 μm long after protargol staining while the cilia on posterior basal bodies with similar length to monokinetid-based ones, about 1 μm long after protargol staining (Figs. 3a, d, 4c, h–j). Lateral ciliary field comprises 13–19 monokinetidal kineties of similar length, each apart 6–13 μm from the anterior cell end, except for the rightmost kinety that commences anteriorly to the second or third kinety of right ciliary field, about 4 μm below the anterior cell end, with the anterior portion curving rightwards before extending towards posterior part; cilia about 2 μm long after protargol staining (Figs. 3b, c, e, 4h, i). Dorsal kinety 29–53 μm long, comprises 25–37 dikinetids, apart about 4 μm from the anterior cell end, about 5 μm from right ciliary field and 13 μm from left ciliary field (Figs. 3c, e, 4j, k). Posterior kinety 21–29 μm long, consists of 11–18 dikinetids, commences posteriorly to lateral ciliary field, with 28–49 μm away from the anterior cell end (Figs. 3c, e, 4j). Cilia of dorsal and posterior kinety are insufficiently stained.

Adoral zone of membranelles comprises 16–18 collar membranelles with cilia about 25–35 μm long, three of which extend into buccal cavity; the longest bases about 30 μm; kinetal structures of membranelles could not be recognized (Figs. 3a–e, 4e–g, h). Single buccal membranelle in buccal cavity, with polykinetid about 20 μm long (Figs. 3b, e, 4e). Endoral membrane comprised of a single row of basal bodies, extends in a semicircle across the peristomial field and right wall of buccal cavity (Figs. 3b, c, 4e, f). Argyrophilic fibers associated with oral apparatus insufficiently impregnated to be observed.

**Fig. 5:**
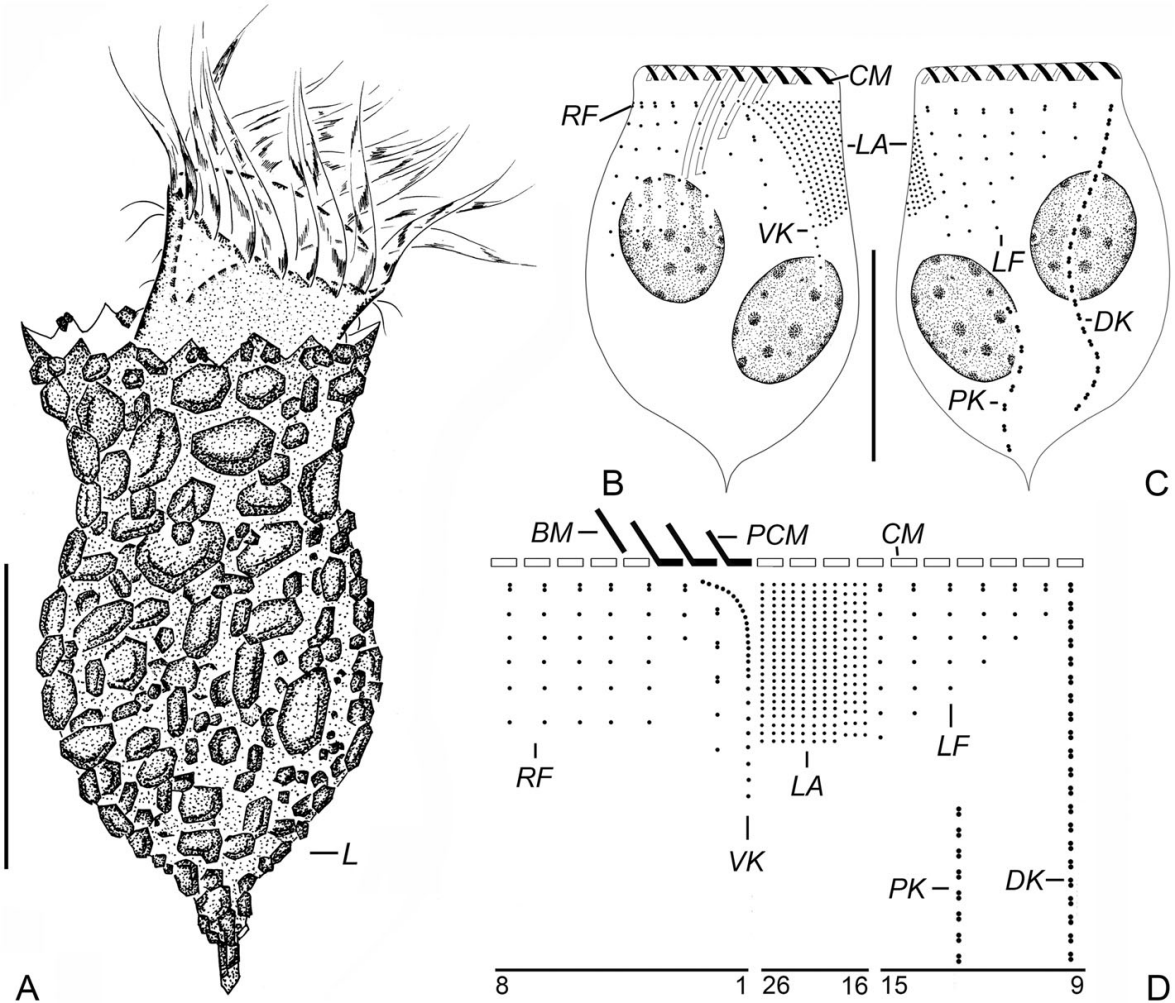
Line drawings of *Tintinnopsis uruguayensis* in vivo (**a**) and after protargol staining (**b**–**d**) (from authors’ own work). **a** Lateral view of a representative individual. **b**, **c** Ventral (**b**) and dorsal (**c**) views of the same specimen, showing ciliary pattern and macronuclear nodules. **d** Kinetal map of a morphostatic specimen. BM, buccal membranelle; CM, collar membranelle; DK, dorsal kinety; L, lorica; LA, lateral ciliary field; LF, left ciliary field; PCM, prolonged collar membranelle; PK, posterior kinety; RF, right ciliary field; VK, ventral kinety. Scale bars = 30 μm (**a**), 15 μm (**b**, **c**)

**Fig. 6:**
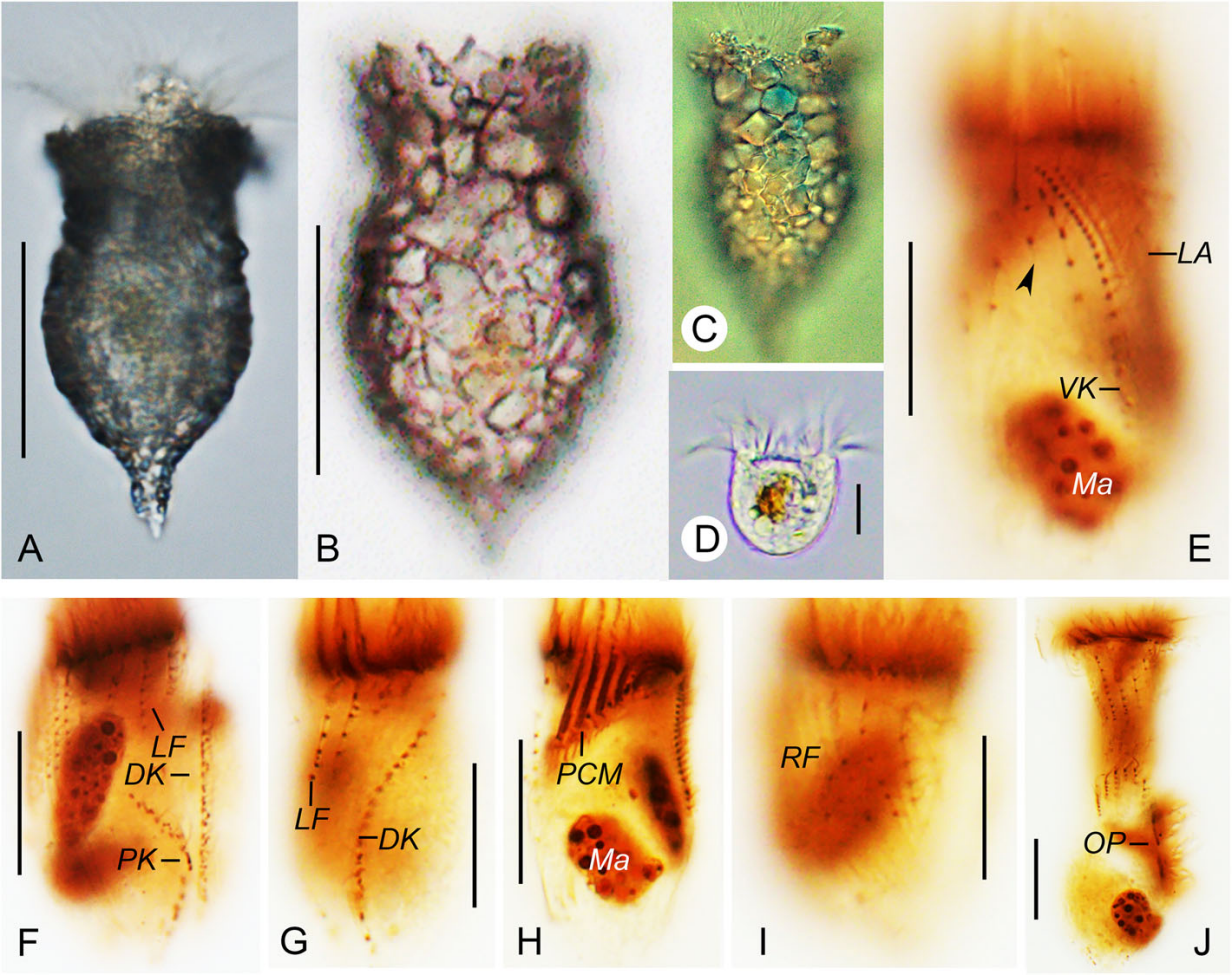
Photomicrographs of *Tintinnopsis uruguayensis* in vivo (**a**–**d**) and after protargol staining (**e**–**j**). **a** Lateral view of a representative individual. **b** Lorica showing a flared collar with jagged rim. **c** Lorica with atypical collar rim. **d** Cell proper escaped from lorica. **e** Ventral side; arrowhead indicates the second short kinety in the right ciliary field. **f** Left ciliary field and dorsal kinety. **g** Dorsal kinety. **h** Prolonged collar membranelles and macronuclear nodules. **i** Right ciliary field. **j** Lateral view of a middle divider. DK, dorsal kinety; LF, left ciliary field; Ma, macronuclear nodule; OP, oral primordium; PK, posterior kinety; RF, right ciliary field; VK, ventral kinety. Scale bars = 30 μm (**a**, **b**), 15 μm (**d**–**k**)

***Tintinnopsis uruguayensis*** **Balech, 1948** (Figs. 5a–d, 6a–j; Table 1).

### Improved diagnosis (based on the type and present populations)

Lorica 50–73 μm long, bullet-like with a flared collar and a posterior process about 8–10 μm long. Opening 22–42 μm in diameter, with an irregular rim. Cell proper obconical when fully extended, size in vivo about 25–50 μm × 20–30 μm. Two macronuclear nodules. On average 18 collar membranelles, of which three or four extend into buccal cavity; one buccal membranelle. Ventral kinety composed of about 20 monokinetids. Right and left ciliary fields consist of about seven kineties each. Lateral ciliary field comprises on average 12 kineties. Dorsal kinety with about 21 dikinetids. Posterior kinety with about eight dikinetids, posterior to lateral ciliary field.

### Deposition of voucher materials

Two protargol slides with voucher specimens were deposited in the Laboratory of Protozoology, Institute of Evolution and Marine Biodiversity, Ocean University of China (registration numbers: BY201811120101 and BY201811120102).

### Redescription based on the Qingdao population

Lorica 50–73 μm long, composed of a flared collar about 15 μm long with a jagged rim, and an ovoidal bowl about 32–52 μm long and 25–41 μm wide (Figs. 5a, 6a–c). Opening diameter 24–42 μm. Region between collar and bowl narrowed, about 17–29 μm in diameter (Figs. 5a, 6a–c). Posterior end projected, about 10 μm long (Figs. 5a, 6a). Wall of lorica heterogeneously agglutinated with mineral particles (Figs. 5a, 6a–c).

Cell proper about 25–50 μm long and 20–35 μm wide from life when fully extended, 25–56 μm long and 18–28 μm wide after protargol staining. Posterior end of cell proper becomes spherical when escaped from lorica (Fig. 6d). Two ellipsoidal (occasionally elongated) macronuclear nodules, 6–14 × 4–10 μm in size after protargol staining; anterior nodule 4–9 μm posteriorly to the anterior cell end after protargol staining (Figs. 5a–c, 6e, h). Micronuclei, striae, tentaculoids, accessory combs, contractile vacuole, cytopyge, and capsules not observed. Locomotion by rotation about main cell axis.

Ventral kinety 14–35 μm long with 17–28 monokinetids, commences anteriorly to first kinety of right ciliary field, about 2 μm posteriorly to the anterior cell end, goes around right ciliary field from the left side and extending parallel to kineties of ciliary field posteriorly (Figs. 5b, d, 6e). Right ciliary field consists of 7–8 kineties, 1–3 μm apart; all kineties commenceabout 2 μm below the anterior cell end, except for the first kinety that commences about 1 μm posteriorly to remaining kineties; the second kinety always shorter with only two or three kinetids; others composed of 6–7 widely spaced monokinetids and one anterior dikinetid, except first kinety comprised of two to four monokinetids and two or three anterior dikinetids; first kinety usually commences below anterior portion of ventral kinety (Figs. 5b, d, 6e, i). Left ciliary field consists of 6–8 kineties about 2 μm away from the anterior cell end, and is composed of one anterior dikinetid and 1–7 monokinetids, with decreasing length from right to left (Figs. 5c, d, 6f, g). The anterior basal bodies of dikinetids in left and right ciliary field bear elongated cilia, about 5 μm long in both live and protargol-stained specimens while the cilia on posterior basal bodies are similar to ones on monokinetids, about 1 μm long after protargol staining (Figs. 5a, 6e–j). Lateral ciliary field begins about 2 μm posteriorly to the anterior end of cell, with 9–16 monokinetidal kineties of similar length, with cilia about 1 μm long after protargol staining (Figs. 5b, d, 6e). Dorsal kinety 21–41 μm long, and consisting of 17–29 dikinetids, begins about 2 μm posteriorly to anterior cell end, about 2 and 3 μm away from right and left ciliary fields, respectively; only the posterior basal body bearing a cilium about 3–5 μm long after protargol staining (Figs. 5c, d, 6f, g). Posterior kinety begins posterior to the middle kinety of the left ciliary field and 12–21 μm apart from the anterior end of cell; 11–22 μm long, consists of 7–9 dikinetids, with only the posterior basal body bearing a cilium about 3–5 μm long after protargol staining (Figs. 5c, d, 6f).

Adoral zone of membranelles consists of 18 or 19 collar membranelles with about 20–25 μm long cilia, three or four of which extend into buccal cavity; the longest bases about 10 μm; polykinetid structures could not be recognized (Figs. 5a–d, 6a, d, h). Single buccal membranelle, with polykinetid about 8 μm long (Figs. 5b, d, 6h). Argyrophilic fibers insufficiently impregnated to be observed. Endoral membrane not recognized. One middle divider was observed with the oral primordium located left of ventral kinety and posterior to the lateral ciliary field (Fig. 6j).

### Neotypification

The neotypes of *Tintinnopsis hemispiralis* and *T. kiaochowensis* are designated because (i) the deposited type materials are unknown; (ii) only lorica features are reported in the original description, while the present redescriptions include also cytological and molecular analyses; and (iii) the type locality of the original populations is Qingdao, East China, with no further details [44]. The type location of the two species is nearby the collection site of the present populations (Meng Bay, Ningde, East China; detailed information provided in ‘Materials and Methods’), thus meeting the requirement of Article 75.3.6 of the International Code of Zoological Nomenclature [51]. Protargol slides containing the neotype specimens were deposited (see ‘Deposition of neotype and other vouched materials’), thus meeting the requirements of Article 75.3.7 of the Code [51]. A neotype is not established for *T. uruguayensis* because the type location corresponds to a different ocean basin [52].

### Sequence comparison and phylogenetic analyses

**Fig. 7:**
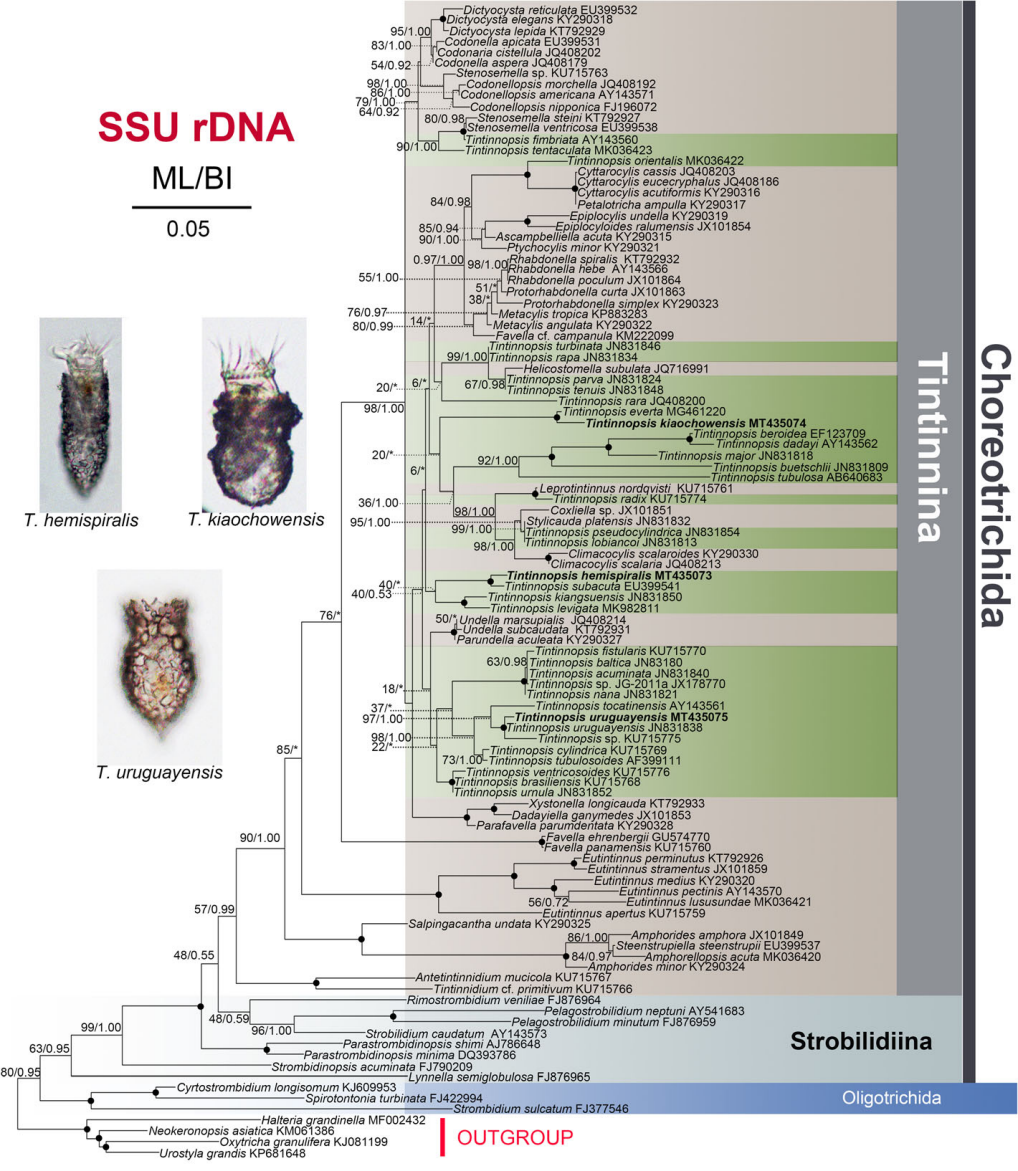
Maximum likelihood (ML) tree inferred from SSU rDNA sequences, showing nodal support for ML and Bayesian Inference (BI) analyses. Newly sequenced species are shown in bold. Asterisks (*) reflect disagreements in topology between the BI and ML trees; black circles reflect fully-supported nodes. The scale bar corresponds to 0.05 substitutions per site

**Fig. 8:**
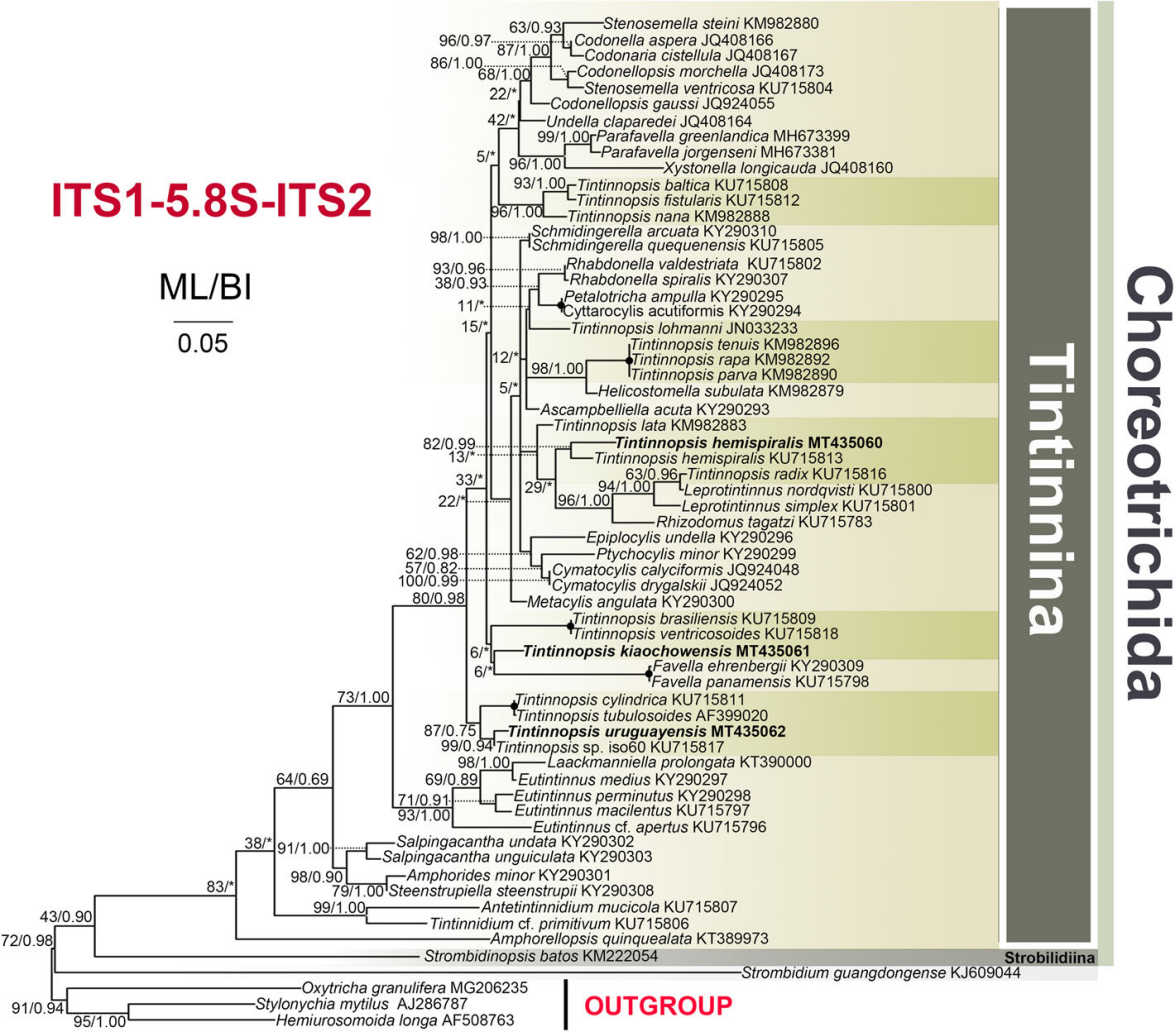
Maximum likelihood (ML) tree inferred from ITS1–5.8S rDNA-ITS2 sequences, showing nodal support for ML and Bayesian Inference (BI) analyses. Newly sequenced species are shown in bold. Asterisks (*) reflect disagreements in topology between the BI and ML trees; black circles reflect fully-supported nodes. The scale bar corresponds to 0.05 substitutions per site

**Fig. 9:**
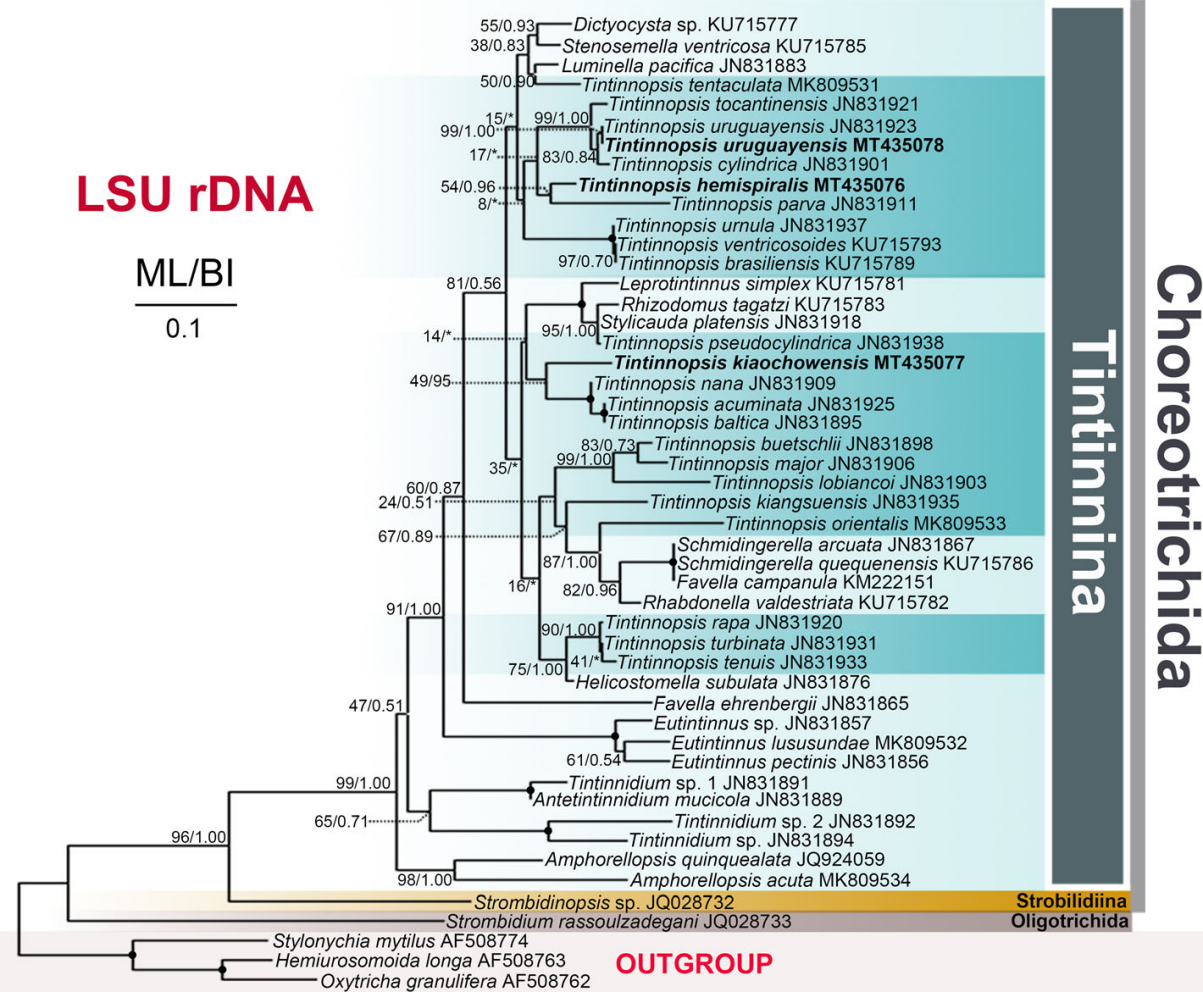
Maximum likelihood (ML) tree inferred from LSU rDNA sequences, showing nodal support for ML and Bayesian Inference (BI) analyses. Newly sequenced species are shown in bold. Asterisks (*) reflect disagreements in topology between the BI and ML trees; black circles reflect fully-supported nodes. The scale bar corresponds to 0.1 substitutions per site

For the three species investigated, the length, G + C content and GenBank accession numbers of the SSU rDNA, ITS1–5.8S rDNA-ITS2 and LSU rDNA sequences are compiled in Table 2. For each of the three loci and concatenated sequences, the topologies of the Maximum Likelihood (ML) and Bayesian Inference (BI) trees were similar and therefore only the ML trees are shown (Figs. 7, 8, 9, S1). *Tintinnopsis hemispiralis* forms a fully-supported clade with *T. subacuta* (EU399541 [53];) based on SSU rDNA; both sequences are 99.3% similar. Based on ITS1–5.8S-ITS2, a sequence previously obtained for this species in Qingdao, China (KU715813 [48];) groups with our sequence, and both are 96.2% similar. *Tintinnopsis kiaochowensis* forms a fully-supported clade with *T. everta* (MG461220 [33];) based on SSU rDNA, and both sequences are 99.0% similar. The newly sequenced population of *T. uruguayensis* forms a fully-supported clade with the North-Atlantic population of the same species, based on both SSU rDNA and LSU rDNA (JN831838 and JN831923 [25];); the two populations are 100% identical in both markers. The concatenated tree (Figure S1, Table S1) shows similar relationships than SSU rDNA, except that Tintinnina were inferred as non-monophyletic. This inference is probably artifactual given the well-known monophyly of this suborder [22, 39].

**Table 2:** DNA sequences obtained in this study

Species	Marker	Length (bp)	GC content (%)	GenBank accession number
*T. hemispiralis*	SSU rDNA	1644	47.45	MT435073
	ITS1–5.8S rDNA-ITS2	493	46.04	MT435060
	LSU rDNA	1704	51.23	MT435076
*T. kiaochowensis*	SSU rDNA	1681	47.06	MT435074
	ITS1–5.8S rDNA-ITS2	418	45.93	MT435061
	LSU rDNA	1695	50.91	MT435077
*T. uruguayensis*	SSU rDNA	2105	47.32	MT435075
	ITS1–5.8S rDNA-ITS2	445	44.97	MT435062
	LSU rDNA	1687	50.50	MT435078

## Discussion

### *Tintinnopsis hemispiralis*

#### Comparison with other populations

The specimens studied here match *Tintinnopsis hemispiralis* in lorica size and shape [44]. The lorica dimensions reported in the original description (length = 88–164 μm, opening diameter = 34–53 μm [44];) overlap with those of our specimens (length = 143–182 μm, opening diameter = 45–59 μm; Table 1). The originally described population and our specimens also match in a lorica composed of a cylindrical, spiraled collar and an obconical bowl (Figs. 1a, 2b–d). One ITS1–5.8S rDNA-ITS2 sequence labeled as *T. hemispiralis* in GenBank [48] presents a relatively high divergence (3.8%) compared to our sequence; conspecificity of both populations cannot be confirmed.

#### Comparison with similar species

Four congeners, namely *Tintinnopsis cochleata* (Brandt, 1906) Laackmann, 1913, *Tintinnopsis directa* Hada, 1932, *Tintinnpsis gracilis* Kofoid and Campbell, 1929, and *Tintinnopsis tubulosoides* Meunier, 1910, are similar to our specimens in an elongated lorica with a spiraled collar. *Tintinnopsis cochleata* differs from our specimens in a sub-hemispherical posterior end and 13 (vs. 3–5) spiral striations in the collar of the lorica [16]. *Tintinnopsis directa* can be separated from our population by a swollen, ovoid bowl and rounded posterior end of the lorica (vs. coniform, pointed [54];). *Tintinnpsis gracilis* differs from our specimens by smaller lorica size (110–135 μm long vs. 143–182 long) and spiraled striation absent in collar portion (vs. 3–5 obvious spiraled striations, see Fig. 2b) [19]. *Tintinnopsis tubulosoides* differs from our specimens in a smaller lorica size (91 μm long and 33 μm in opening diameter, based on the illustration included in the original description) and two (vs. 7–11) macronuclear nodules [55]. For these species, rDNA sequences have been reported only for *T. tubulosoides* (AF399111–AF399020 [56];), which shows a distant relationship to *T. hemispralis* (Figs. 7, 8).

Regarding cell features, *T. hemispiralis* resembles *Tintinnopsis subacuta* Jörgensen, 1899 in having a ventral kinety associated with the extraordinarily long ciliary tuft that extends outside of the lorica [50]. Both species are also similar in having multiple moniliform macronuclear nodules [50], which differ from the common finding of only two macronuclear nodules in other *Tintinnopsis* species (e.g., [22, 27, 34]). The two species cluster together based on SSU rDNA (Fig. 7), which suggests that the ciliary tuft and multiple moniliform macronuclear nodules are synapomorphies of this clade and may be important for a future reclassification of *Tintinnopsis* species. Despite the close relationship between *T. hemispiralis* and *T. subacuta*, the latter can be distinguished from our specimens by a lorica with a swollen, ovoid (vs. obconical) bowl in the original description [57] and the micrograph of the sequenced specimen [53]. The SSU rDNA divergence for both species, although small (0.7%), is consistent with interspecific variation in this conserved marker [25].

### *Tintinnopsis kiaochowensis*

#### Comparison with type population

The specimens studied here match *Tintinnopsis kiaochowensis* in lorica size and shape [44]. The lorica dimensions reported in the original description (length = 95–108 μm, opening diameter = 30–52 μm [44];) overlap with those of our specimens (length = 79–112 μm, opening diameter = 44–71 μm; Table 1). Our specimens also resemble to those originally described in a lorica with a cylindrical collar and an ellipsoidal bowl with a constricted connection. However, our specimens differ from the original population in the rounded posterior end of the lorica (vs. obconical) and in the agglutinated particles forming horizontal rows on the collar (vs. both on collar and bowl) [44].

#### Comparison with similar species

*Tintinnopsis kiaochowensis* differs from other *Tintinnopsis* species by its peculiar lorica shape, i.e. swollen bowl divided from a non-flaring collar by a constriction. Compared to our specimens, the most similar species is *Tintinnopsis compressa* Daday, 1887. However, *T. compressa* can be separated from our specimens by having a smaller lorica size (45 vs. 79–112 μm in length; 26 vs. 44–71 μm in opening diameter), a flared lorica collar (vs. not flared), and a less obvious constriction between the lorica collar and bowl [18].

*Tintinnopsis kiaochowensis* is similar to *Tintinnopsis everta* Kofoid and Campbell, 1929 based on SSU rDNA (Fig. 7) and cytological characters [33], including: (i) elongated anterior portion of the ventral kinety, which forms a curvature above the third, occasionally the fourth, kinety of the right ciliary filed; (ii) elongated anterior portion of the rightmost kinety of lateral ciliary field, which forms a curvature above the second or third kinety of the right ciliary field (with the ventral kinety in between); and (iii) first four to six kineties of the right ciliary field very widely spaced. However, unique cytological features observed in *T. everta* (the large distance between the collar membranelles and the somatic ciliary fields as well as the position of the posterior kinety [33];) are not present in *T. kiaochowensis*. Both species also show a different lorica morphology (campanulate lorica with a funnel-shaped collar vs. ellipsoidal bowl and non-flaring collar, respectively) and interspecies-level divergence in SSU rDNA (1% [25];).

### *Tintinnopsis uruguayensis*

#### Comparison with other populations

This species was first described by Balech [52] based on the lorica features of specimens collected in the Southwest Atlantic Ocean. The lorica dimensions reported in the original description (length = 54–63 μm, opening diameter = 22–27 μm [52];) overlap with those of our specimens (length = 50–73 μm, opening diameter = 24–42 μm; Table 1), and both populations match in the characteristic bullet-like shape with a flared collar and a posterior process. Our population presents no divergence in SSU rDNA and LSU rDNA when compared against Long Island Sound specimens of similar lorica features [25].

#### Comparison with similar species

In terms of a small, bullet-like lorica, three congeners, namely *Tintinnopsis baltica* Brandt, 1896, *Tintinnopsis fimbriata* Meunier, 1919, and *Tintinnopsis meunieri* Kofoid and Campbell, 1929, can be compared to our population. *Tintinnopsis baltica* has a similar lorica shape, but can be separated from *T. uruguayensis* by the absence (vs. presence) of a protruding posterior end [58]. Laval-Peuto & Brownlee [59] provided a diagram of the ciliary pattern of *T. baltica*, which is similar to our specimens in the number of kineties in the right, left, and lateral ciliary fields and the presence of only 2–3 kinetids in the second kinety of right ciliary field, but differs in a shorter ventral kinety. The distant phylogenetic relationship between *T. uruguayensis* and *T. baltica* based on SSU rDNA and LSU rDNA (Figs. 7, 9) also separates both species. *Tintinnopsis fimbriata* differs from *T. uruguayensis* by a shorter collar (10 μm vs. up to 20 μm) and a wider bowl (40–50 μm vs. 25–41 μm) [60]. Based on cytological data [27], *T. fimbriata* also differs from the latter in having less kineties in the left ciliary field (4–6 vs. up to 9) and lateral ciliary field (11–14 vs. up to 17). The SSU rDNA sequence labeled as *T. fimbriata* in GenBank (Fig. 7) has been considered a misidentification [39] and is thus not considered in this comparison. *Tintinnopsis meunieri* differs from *T. uruguayensis* in a larger opening diameter (60 μm vs. 24–42 μm) [19].

## Conclusion

*Tintinnopsis hemispiralis*, *T. kiaochowensis* and *T. uruguayensis* show hard, fully agglomerated loricae and the most complex pattern of somatic ciliature known for the genus, i.e. a right, left and lateral ciliary field as well as a ventral, dorsal and posterior kinety [22]. However, the three species show differences in the lorica outline and the number, structure and arrangement of somatic kineties (Figs. 1, 2, 3, 4, 5 and 6; Table 1), and species-level divergence in rRNA genes [25, 26]. Their distant position and intertwining with other genera in phylogenetic trees (Figs. 7, 8, 9) confirm, once again, the non-monophyly of the genus *Tintinnopsis* [22, 38, 39].

*Tintinnopsis* cannot be revised at present, as its type species and most other tintinnine species have not been studied cytologically or genetically [23]. Our work is important to increase the number of tintinnine species investigated with modern methods, which also helps in identifying potential synapomorphies for future taxonomic rearrangements. Our data show the potential taxonomic relevance of (i) details of the somatic ciliary pattern, including the anterior parts of the ventral kinety and the rightmost kinety of the lateral ciliary field [33]; and (ii) the presence of a ciliary tuft and multiple moniliform macronuclear nodules. Our paper contributes important information on the non-monophyletic *Tintinnopsis* and it thus helps to fill the gaps in modern tintinnine taxonomy.

## Methods

### Sample collection and morphological analysis

**Fig. 10:**
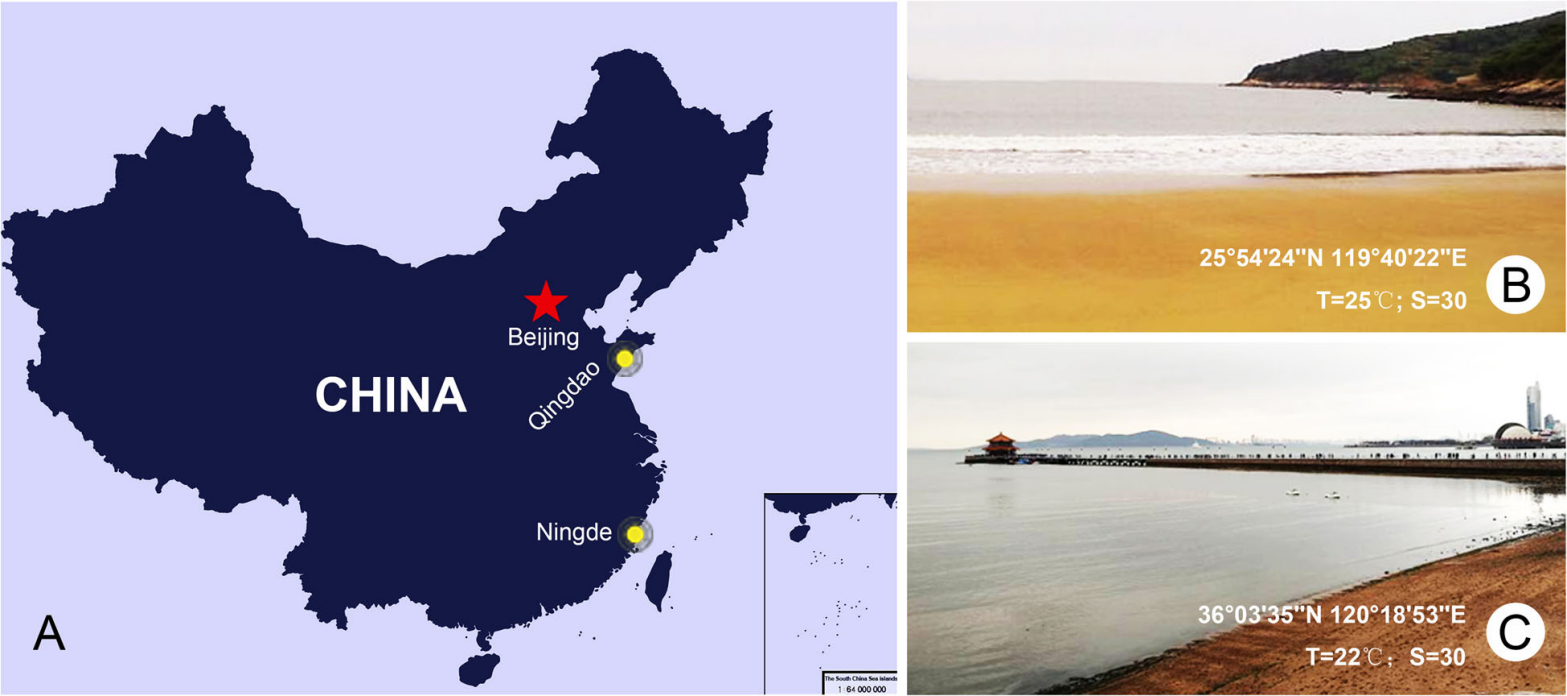
Sampling sites. **a** Map of China with sample sites (yellow circles), downloaded from the open-access website: www.osgeo.cn. **b**, **c** Photographs of Meng Bay and coast of Qingdao, respectively

*Tintinnopsis hemispiralis* and *Tintinnopis kiaochowensis* were collected from surface coastal waters in Meng Bay, Ningde, Fujian Province, China (25°54′24″N 119°40′22″E; temperature = 25 °C; salinity = 30) on May 28, 2018 (Fig. 10a, b); *Tintinnopsis uruguayensis* was collected from surface coastal waters off Qingdao, Shandong Province, China (36°03′35″N 120°18′53″E; temperature = 22 °C; salinity = 30) on November 12, 2018 (Fig. 10a, c).

Live cells were observation and protargol staining were performed as Bai et al. [31]. Loricae were measured from living cells at magnifications 100–400× with accuracy 1 μm. Identifications were based on original descriptions [44, 52] and other tintinnine bibliography mentioned above. Terminology and classification follow Agatha & Riedel-Lorjé [61] and Adl et al. [62], respectively.

### DNA extraction, PCR amplification and sequencing

Because most tintinnine species are not amenable to culture, clonal cultures could not be established. Thus, we applied common criteria to verify that field-isolated specimens were not confounded with other species (e.g. as done by Gruber et al. [33]): the three species were distinguished by careful evaluation of their morphological features and lorica size in vivo, and the absence of potentially confounding, co-occurring species was confirmed with further analyses of loricae and protargol-stained cells. For each species, a single specimen was isolated at 400× magnification and washed five times with 0.22-μm filtered sample water. DNA extraction, PCR amplification and sequencing were done as detailed by Bai et al. [31], except for some of the primers utilized. PCR amplification of the SSU rDNA was performed with the primers 82F (5′-GAA ACT GCG AAT GGC TC-3′ [63];) and either 5.8 s-R (5′-CTG ATA TGC TTA AGT TCA GCG G-3′ [64];) for *Tintinnopsis uruguayensis* or 18 s-R (5′-TGA TCC TTC TGC AGG TTC ACC TAC-3′ [65];) for the other two species. A fragment containing the ITS1, 5.8S rDNA and ITS2 regions was amplified with the primers 5.8 s-F (5′-GTA GGT GAA CCT GCG GAA GGA TC-3′) and 5.8 s-R (5′-CTG ATA TGC TTA AGT TCA GCG G-3′) [64].

Sequences were assembled and analysed as reported before [31]. In brief, phylogenetic analyses were done separately for SSU rDNA, ITS1–5.8S rDNA-ITS2 and LSU rDNA, as well as after concatenating the three sequence markers. The analyse incorporated additional ciliate sequences were obtained from GenBank and used *Halteria grandinella* and hypotrichs as outgroup taxa. Sequences were aligned with Muscle 3.7 [66]. Maximum likelihood analyses were done with RAxML v. 8 [67], using the GTRGAMMA model and 1000 bootstraps. Bayesian Inference analyses were done with MrBayes v.3.2.6 [68], using the GTR + I + Γ model, 6000,000 generations with a sample frequency of 100 generations and a burn-in of 6000 trees. Estimates of sequence similarity were done in MEGA 7.0 [69].

## Supplementary Information

**Additional file 1: Figure S1.** Maximum likelihood (ML) tree inferred from concatenated rDNA loci (SSU rDNA, ITS1–5.8S-ITS2 and LSU rDNA) showing nodal support for ML and BI analyses. Newly sequenced species, i.e., *Tintinnopsis hemispiralis*, *T. kiaochowensis*, and *T. uruguayensis* are shown in bold. See Table S1 for GenBank accession numbers. All species possess SSU rDNA; species including ITS1–5.8S-ITS2 were marked with red stars; species including LSU rDNA were marked with green circles. Species with no marks include the three loci. Asterisks (*) reflect disagreements in topology between the BI and ML trees; black circles reflect fully-supported nodes. The scale bar corresponds to 0.1 expected substitutions per site. **Table S1.** List of sequences of concatenated tree. Newly sequenced species, i.e., *Tintinnopsis hemispiralis*, *T. kiaochowensis*, and *T. uruguayensis* are shown in bold.

## Data Availability

Sequence data is available in GenBank (Accession Numbers: MT435060–MT435062, MT435073–MT435078). Permanent slides containing the protargol-stained specimens of *Tintinnopsis hemispiralis*, *T. kiaochowensis,* and *T. uruguayensis* with registration numbers of BY201805280101, BY201805280102, BY201805280201, BY201805280202, BY201811120101, and BY201811120102 are Laboratory of Protozoology, Institute of Evolution and Marine Biodiversity, Ocean University of China.

## Abbreviations

BM: Buccal membranelle
CM: Collar membranelle
CV: Coefficient of variation in %
DK: Dorsal kinety
EM: Endoral membrane
LA: Lateral ciliary field
LF: Left ciliary field
M: Median
Ma: Macronuclear nodules
Max: Maximum
Mean: Arithmetic mean
Min: Minimum
N: Number of specimens examined
PCM: Prolonged collar membranelles
PK: Posterior kinety
RF: Right ciliary field
SD: Standard deviation
VK: Ventral kinety
