# Supplementary material for: Three redescriptions in Tintinnopsis (Protista: Ciliophora: Tintinnina) from coastal waters of China, with cytology and phylogenetic analyses based on ribosomal RNA genes
Source: BMC Microbiol. 2020 Dec 14;20:374. doi: 10.1186/s12866-020-02057-2 (PMC7737391; doi:10.1186/s12866-020-02057-2)
Supplement: Supplementary file 1 — Additional file 1: Figure S1. Maximum likelihood (ML) tree inferred from concatenated rDNA loci (SSU rDNA, ITS1–5.8S-ITS2 and LSU rDNA) showing nodal support for ML and BI analyses. Newly sequenced species, i.e., Tintinnopsis hemispiralis, T. kiaochowensis, and T. uruguayensis are shown in bold. See Table S1 for GenBank accession numbers. All species possess SSU rDNA; species including ITS1–5.8S-ITS2 were marked with red stars; species including LSU rDNA were marked with green circles. Species with no marks include the three loci. Asterisks (*) reflect disagreements in topology between the BI and ML trees; black circles reflect fully-supported nodes. The scale bar corresponds to 0.1 expected substitutions per site. Table S1. List of sequences of concatenated tree. Newly sequenced species, i.e., Tintinnopsis hemispiralis, T. kiaochowensis, and T. uruguayensis are shown in bold. [file 12866_2020_2057_MOESM1_ESM.pdf]

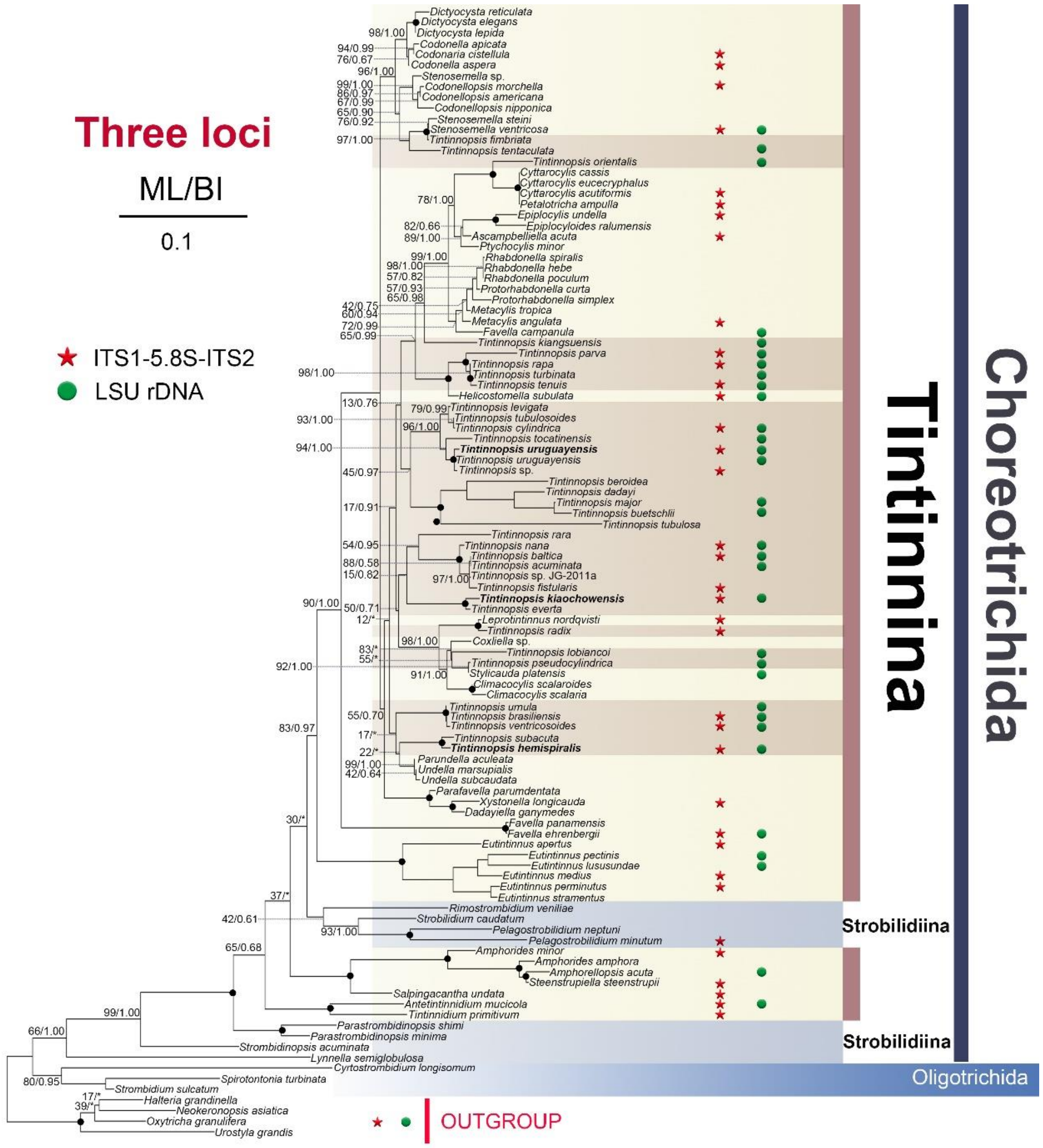

Figure S1. Maximum likelihood (ML) tree inferred from concatenated rDNA loci (SSU rDNA, ITS1-5.8S-ITS2 and LSU rDNA) showing nodal support for ML and BI analyses. Newly sequenced species, i.e., *Tintinnopsis hemispiralis*, *T. kiaochowensis*, and *T. uruguayensis* are shown in bold. See Table S1 for GenBank accession numbers. All species possess SSU rDNA; species including ITS1-5.8S-ITS2 were marked with red stars; species including LSU rDNA were marked with green circles. Species with no marks include the three loci. Asterisks (*) reflect disagreements in topology between the BI and ML trees; black circles reflect fully-supported nodes. The scale bar corresponds to 0.1 expected substitutions per site.

Table S1. List of sequences of concatenated tree. Newly sequenced species, i.e., *Tintinnopsis hemispiralis*, *T. kiaochowensis*, and *T. uruguayensis* are shown in bold.

Species in concatenated rDNA	SSU rDNA	ITS1-5.8S-ITS2	LSU rDNA

	GB accession number	GB accession number	GB accession number
Amphorellopsis acuta	MK036420		MK809534
Amphorides amphora	JX101849		
Amphorides minor	KY290324	KY290301	
Antetintinnidium mucicola	KU715767	KU715807	JN831889
Ascampbelliella acuta	KY290315	KY290293	
Climacocylis scalaria	JQ408213		
Climacocylis scalaroides	KY290330		
Codonaria cistellula	JQ408202	JQ408167	
Codonella apicata	EU399531		
Codonella aspera	JQ408179	JQ408166	
Codonellopsis americana	AY143571		
Codonellopsis morchella	JQ408192	JQ408173	
Codonellopsis nipponica	FJ196072		
Coxliella sp.	JX101851		
Cyrtostrombidium longisomum	KJ609953		
Cyttarocylis acutiformis	KY290316	KY290294	
Cyttarocylis cassis	JQ408203		
Cyttarocylis eucecryphalus	JQ408186		
Dadayiella ganymedes	JX101853		
Dictyocysta elegans	KY290318		
Dictyocysta lepida	KT792929		
Dictyocysta reticulata	EU399532		
Epiplocyloides ralumensis	JX101854		
Epiplocylis undella	KY290319	KY290296	
Eutintinnus apertus	KU715759	KU715796	
Eutintinnus lususundae	MK036421		MK809533
Eutintinnus medius	KY290320	KY290297	
Eutintinnus pectinis	AY143570		JN831856
Eutintinnus perminutus	KT79292	KY290298	
Eutintinnus stramentus	JX101859		
Favella campanula	KM222099		KM222151
Favella ehrenbergii	GU574770		JN831865
Favella panamensis	KU715760		
Halteria grandinella	MF002432		
Helicostomella subulata	JQ716991	KM982879	JN831876
Leprotintinnus nordqvisti	KU715761	KU715800	
Lynnella semiglobulosa	FJ876965		
Metacylis angulata	KY290322	KY290300	
Metacylis tropica	KP883283		
Neokeronopsis asiatica	KM061386		
Novistrombidium apsheronicum	FJ876958		
Oxytricha granulifera	KJ081199	MG206235	AF508762

Parafavella parumdentata	KY290328		
Parastrombidinopsis minima	DQ393786		
Parastrombidinopsis shimi	AJ786648		
Parundella aculeata	KY290327		
Pelagostrobilidium minutum	FJ876959		
Pelagostrobilidium neptuni	AY541683		
Petalotricha ampulla	KY290317	KY290295	
Protorhabdonella curta	JX101863		
Protorhabdonella simplex	KY290323		
Ptychocylis minor	KY290321	KY290299	
Rhabdonella hebe	AY143566		
Rhabdonella poculum	JX101864		
Rhabdonella spiralis	KT792932	KY290307	
Rimostrombidium veniliae	FJ876964		
Salpingacantha undata	KY290325		
Spirotontonia turbinata	FJ422994		
Steenstrupiella steenstrupii	EU399537	KY290308	
Stenosemella sp.	KU715763		
Stenosemella steini	KT792927	KM982880	
Stenosemella ventricosa	EU399538	KU715804	KU715785
Strobilidium caudatum	FJ377546		
Strombidinopsis acuminata	FJ790209		
Strombidium sulcatum	AY143573		
Stylicauda platensis	JN831832		JN831918
Tintinnidium primitivum	KU715766	KU715806	
Tintinnopsis acuminata	JN831840		JN831925
Tintinnopsis baltica	JN83180	KU715808	JN831895
Tintinnopsis beroidea	EF123709		
Tintinnopsis brasiliensis	KU715768	KU715809	KU715789
Tintinnopsis buetschlii	JN831809		JN831898
Tintinnopsis cylindrica	KU715769	KU715811	JN831901
Tintinnopsis dadayi	AY143562		
Tintinnopsis everta	MG461220		
Tintinnopsis fimbriata	AY143560		
Tintinnopsis fistularis	KU715770	KU715812	
Tintinnopsis hemispiralis	**MT435073**	**MT435060**	**MT435076**
Tintinnopsis kiaochowensis	**MT435074**	**MT435061**	**MT435077**
Tintinnopsis kiangsuensis	JN831850		JN831935
Tintinnopsis levigata	MK982811		
Tintinnopsis lobiancoi	JN831813		JN831903
Tintinnopsis major	JN831818		JN831906
Tintinnopsis nana	JN831821	KM982888	JN831909
Tintinnopsis orientalis	MK036422		MK809532
Tintinnopsis parva	JN831824	KM982890	JN831911

Tintinnopsis pseudocylindrica	JN831854		JN831938
Tintinnopsis radix	KU715774	KU715816	
Tintinnopsis rapa	JN831834	KM982892	JN831920
Tintinnopsis rara	JQ408200		
Tintinnopsis sp.	KU715775	KU715817	
Tintinnopsis sp. JG-2011a	JX178770		
Tintinnopsis subacuta	EU399541		
Tintinnopsis tentaculata	MK036423		MK809531
Tintinnopsis tenuis	JN831848	KM982896	JN831933
Tintinnopsis tocatinensis	AY143561		JN831921
Tintinnopsis tubulosa	AB640683		
Tintinnopsis tubulosoides	AF399111	AF399020	
Tintinnopsis turbinata	JN831846		JN831931
Tintinnopsis urnula	JN831852		JN831937
Tintinnopsis uruguayensis	**MT435075**	**MT435062**	**MT435078**
Tintinnopsis uruguayensis	JN831838		JN831923
Tintinnopsis ventricosoides	KU715776	KU715818	KU715793
Undella marsupialis	JQ408214		
Undella subcaudata	KT792931		
Urostyla grandis	KP681648		
Xystonella longicauda	KT792933	JQ408160	
